# Metagenomic characterisation of additional and novel avian viruses from Australian wild ducks

**DOI:** 10.1038/s41598-020-79413-9

**Published:** 2020-12-17

**Authors:** Jessy Vibin, Anthony Chamings, Marcel Klaassen, Soren Alexandersen

**Affiliations:** 1 Geelong Centre for Emerging Infectious Diseases, Geelong, VIC 3220 Australia; 2 School of Medicine, Deakin University, Geelong, VIC 3220 Australia; 3 Centre for Integrative Ecology, Deakin University, Waurn Ponds, VIC 3216 Australia; 4 Barwon Health, Geelong, VIC 3220 Australia

**Keywords:** Microbiology, Virology

## Abstract

Birds, notably wild ducks, are reservoirs of pathogenic and zoonotic viruses such as influenza viruses and coronaviruses. In the current study, we used metagenomics to detect and characterise avian DNA and RNA viruses from wild Pacific black ducks, Chestnut teals and Grey teals collected at different time points from a single location. We characterised a likely new species of duck aviadenovirus and a novel duck gyrovirus. We also report what, to the best of our knowledge, is the first finding of an avian orthoreovirus from Pacific black ducks and a rotavirus F from Chestnut teals. Other viruses characterised from the samples from these wild ducks belong to the virus families *Astroviridae, Caliciviridae* and *Coronaviridae.* Some of the viruses may have potential cross-species transmissibility, while others indicated a wide genetic diversity of duck viruses within a genus. The study also showed evidence of potential transmission of viruses along the East Asian—Australasian Flyway; potentially facilitated by migrating shorebirds. The detection and characterisation of several avian viruses not previously described, and causing asymptomatic but potentially also symptomatic infections suggest the need for more virus surveillance studies for pathogenic and potential zoonotic viruses in wildlife reservoirs.

## Introduction

Birds, notably wild ducks, can be reservoirs for zoonotic viruses including, but not limited to, influenza viruses^1,2^ and coronaviruses^3,4^. These viruses could become potential threats to other wild animals, poultry and possibly humans under the right set of circumstances^2^. Virome (virus community) analysis of potential reservoir bird hosts is expected to unveil the role of viruses causing acute or long-term asymptomatic infections and the diversity and range of viruses that infect wild birds. Virome analysis is also expected to aid in understanding the transmission and possible role of viruses on host health in the wild. Metagenomics of viruses, utilises the power of NGS for the discovery, analysis and characterisation of viruses, thereby enabling virome analysis and providing insight into viruses for which these birds act as a natural reservoir. The use of the metagenomic technique in bird samples can lead to the determination of the virome from an individual bird or a population, and the identification of various novel avian viruses^3,5,6^. Nevertheless, only a few studies have explored the viruses circulating among wild ducks in Australia^3–6^.

In the current study, we collected fresh duck faecal samples from a single location at various time points. Metagenomics of viruses was carried out to detect and characterise avian viruses present in the sample of Australian wild ducks using a protocol optimised in our laboratory and described earlier^3^. We previously focused on avian parvoviruses and picornaviruses from these duck samples as they were found to be abundant compared to other avian viruses^6^. We also reported a reassorted low pathogenicity H9N2 avian influenza virus with intercontinental gene segments in Chestnut teal samples collected on August 2018^1^. It was the first report of an H9N2 avian influenza virus in resident wild birds in Australia^1^. In the current study, we focus on the sequence characterisation of additional avian DNA and RNA viruses from multiple virus families that were present in the Australian wild ducks. We aimed to determine the abundance of the different viruses in Australian wild ducks and the factors that might influence the ecology of these viruses in relation to host species.

## Results

We have previously described viruses belonging to the virus families *Parvoviridae* and *Picornaviridae* from our duck samples as they had the most abundant virus reads generated^6^. Avian viruses belonging to virus families other than *Parvoviridae* and *Picornaviridae* are described here and were detected and characterised in samples from Pacific black ducks (PBD12.16, PBD05.18 and PBD08.18), Chestnut teals (CT08.18 and CT11.18) and Grey teals (GT11.18) (Table 1)*.* From the Chestnut teal (CT05.18) and Wood duck (WD08.18) samples collected in May and August 2018, respectively, no other avian viruses were detected other than those previously described viruses belonging to *Parvoviridae* and *Picornaviridae*^6^.

**Table 1:** Avian viruses detected and characterised from the duck samples.

Sample	Avian viruses detected and characterised
**Pacific black duck**	
PBD12.16	Aviadenovirus (PBDAdV/PBD12.16)
	Calicivirus (PBDCV/PBD12.16)
PBD05.18	Avastrovirus (PBDAstV/PBD05.18)
PBD08.18	Avian orthoreovirus (PBDORV/PBD08.18)
	Rotavirus G (PBDRVG/PBD08.18)
**Chestnut teal**	
CT08.18	Gammacoronavirus
	Rotavirus F (CTRVF/CT08.18)
CT11.18	Avastrovirus (CTAstV/CT11.18)
**Grey teal**	
GT11.18	Gyrovirus (GTGV/GT11.18)

A total of nine viruses were characterised from the duck samples, of which eight of them are described in the current study in detail. Viruses characterised previously from these duck samples are published earlier^1,3,4,6^.

### DNA viruses characterised from the duck samples

Two DNA viruses, in addition to the parvoviruses already described^6^, were detected and characterised from the duck samples, one belonging to the virus family *Adenoviridae* and another to *Anelloviridae.*

#### Aviadenovirus from Pacific black duck (PBDAdV/PBD12.16)

**Figure 1:**
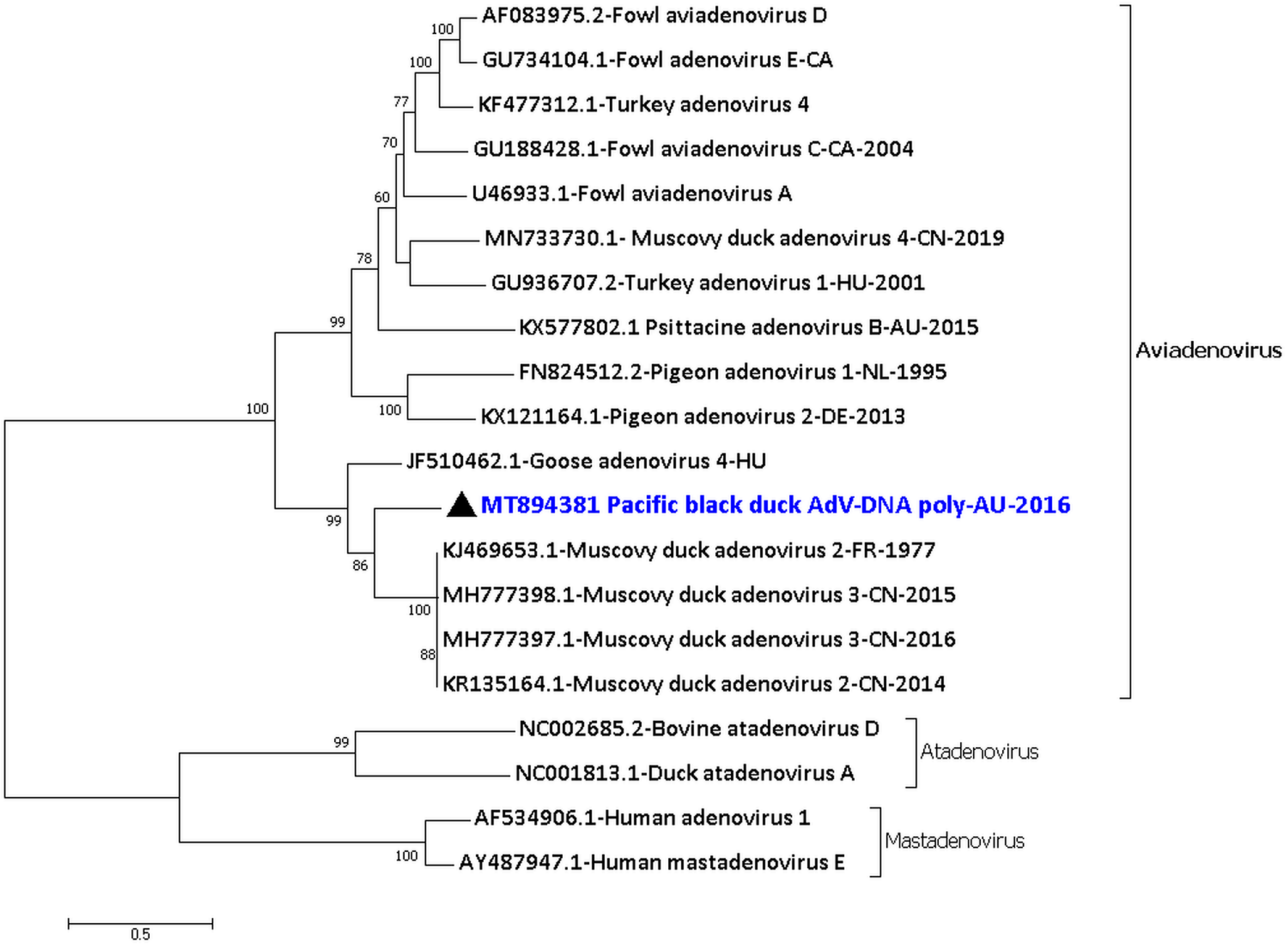
Phylogenetic analysis of the amino acid sequences of the DNA polymerase of Pacific black duck aviadenovirus (PBDAdV/PBD12.16). The amino acid sequences were aligned and analysed by using the maximum likelihood method based on the LG + G model^72^ in MEGA7^71^ with a bootstrapping of 1000 replicates. The analysis involved 20 amino acid sequences and all positions containing gaps and missing data were eliminated. The final dataset contained a total of 936 amino acid positions. The numbers at the nodes represent bootstrap values and only bootstrap values at or above 60% are shown. Pacific black aviadenovirus is shown in blue and marked with a black triangle.

A brief account of this avian virus detected in this sample was provided earlier^3^. However, more nucleotide sequences were generated by resequencing the sample at a greater depth. A total of 37,832 nucleotides (out of ~ 43 kb) of adenovirus sequence was generated from seven consensus sequences of the virus with a coverage ranging from 2 to 4456 (Supplementary material 1 Row 1–7 and Supplementary material 2 Fig. S1). The seven consensus sequences of the Pacific black duck aviadenovirus encoded the dUTPase, ORF52, ORF2, ORF14, ORF12, IVa2, DNA polymerase, pTP, partial 52 K, complete pIIIa, penton base protein, pVII, pX, minor capsid protein VI, hexon, protease, DNA binding protein, 100 K, 22 K, 33 K, pVIII, U-exon, partial fibre, complete ORF22, ORF20, ORF56, ORF19 (lipase), partial ORF54, partial ORF19B and complete ORF53. The amino acid sequences of the DNA polymerase showed that the PBDAdV/PBD12.16 was only 81.9% (1028/1255 amino acids) identical to the closest sequence in NCBI which is a Muscovy duck adenovirus 2 from France in the year 1977^7^ (KJ469653.1), and only 81.5% (1024/1255 amino acids) identical to JF510462.1 Goose adenovirus 4 from Hungary^8^ (Fig. 1). The species demarcation criteria of the genus *Aviadenovirus* by ICTV is based on DNA polymerase phylogenetic distance (> 5–15% distance matrix), genomic organisation, RFLP analysis, host range, pathogenicity, cross-neutralization and ability to recombine^9^. From the PBDAdV/PBD12.16 consensus sequences generated here and based on the DNA polymerase phylogenetic analysis and host species; this virus can be considered potentially as a new species. BLASTP analysis of these protein sequences showed that some of them are more identical but still distant to their counterparts expressed by the KJ469653.1 Muscovy duck adenovirus 2 or the KR135164.1 Muscovy duck adenovirus 2, while others to that of JF510462.1 Goose adenovirus 4 (Supplementary Material 1 Column J).

From the generated consensus sequences, 18 genes have been identified (IVa2–fibre) to be located in the central region of the Pacific black duck adenovirus genome. Thirty genes or ORFs (out of 34–36 genes) were found in total from the seven consensus sequences of the virus. The motifs and other sequence or amino acid features found such as bipartite nuclear localisation signals have been given in the Supplementary material 1 Column K.

#### Gyrovirus from Grey teal (GTGV/GT11.18)

**Figure 2:**
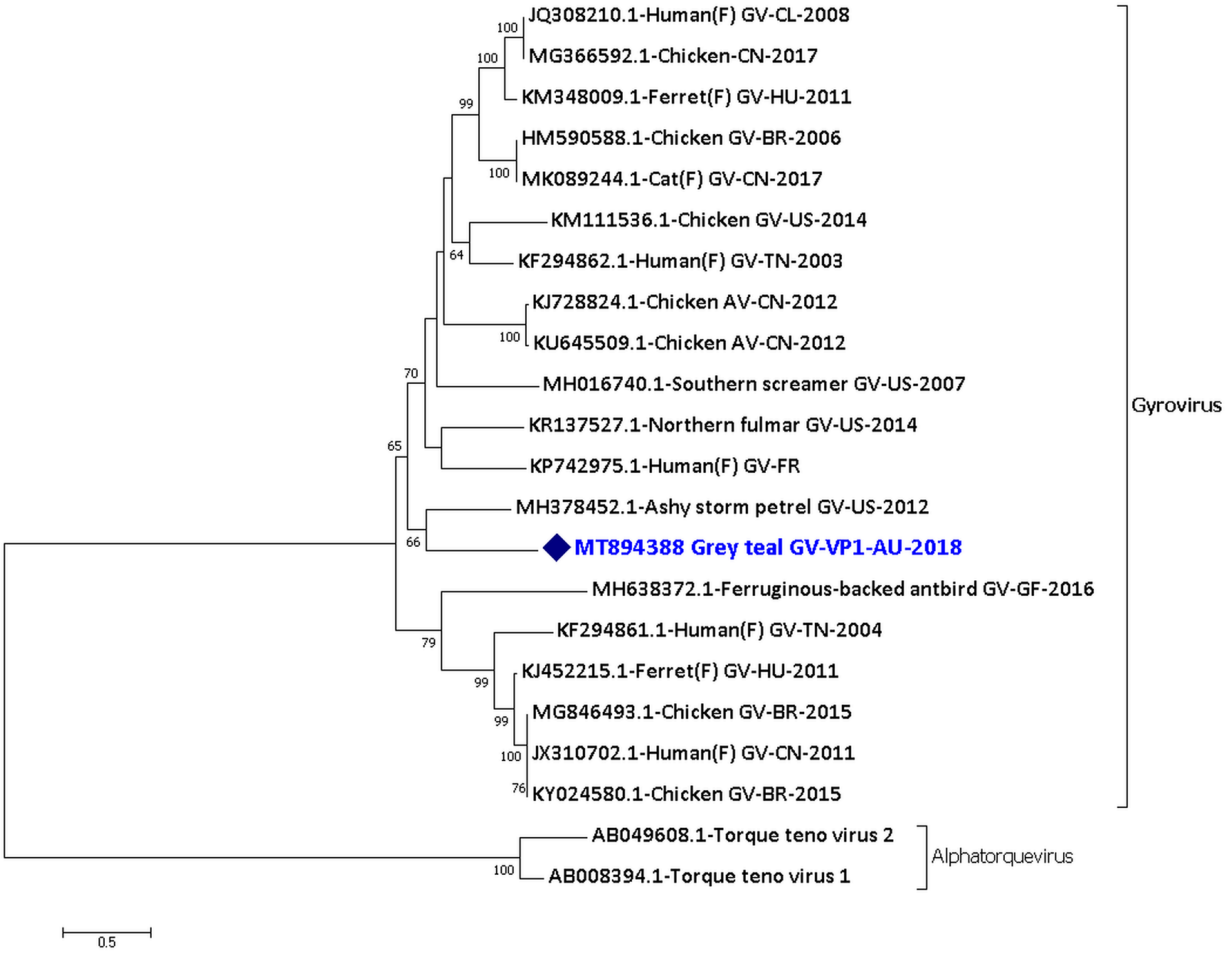
Phylogenetic analysis of the amino acid sequences of the VP1 protein of Grey teal gyrovirus (GTGV/GT11.18). The amino acid sequences were aligned and analysed using the maximum likelihood method based on the WAG + G + I + F model^73^ in MEGACC^74^ with a bootstrapping of 1000 replicates. The analysis involved 22 amino acid sequences and all positions containing gaps and missing data were eliminated. The final dataset contained a total of 233 amino acid positions. The numbers at the nodes represent bootstrap values and only bootstrap values at or above 60% are shown. Grey teal gyrovirus is shown in blue and marked with a blue diamond. (F) shows faecal samples.

A novel gyrovirus was found in the single Grey teal sample (GT11.18). Real-time PCR amplification and Sanger sequencing were carried out and were successful for the Grey teal gyrovirus for the region from 264th to 761st nucleotide. A near-complete genome of 1549 nucleotides long consensus sequences encoding complete VP1, VP2 and hypothetical VP3 protein of GTGV/GT11.18 was generated from the NGS reads of the sample with a coverage ranging from 2 to 105 (Supplementary material 1 Row 47). The phylogenetic analysis of the VP1 protein sequence of the Grey teal gyrovirus shows that it is distantly related to other currently known gyroviruses (Fig. 2). Gyroviruses were recently classified into the family *Anelloviridae,* and the species demarcation criteria of some of the genera in the family *Anelloviridae* are based on ORF1/VP1 (capsid) protein with a nucleotide divergence of > 35%^10^. Hence, based on the phylogenetic analysis of the VP1 protein sequence the Grey teal gyrovirus can be considered as a new virus species. The GTGV/GT11.18 was relatively most identical, but still distant, to MH378452.1 gyrovirus from Ashy storm petrel faecal sample from the US in 2012 with 55.2% (159/288 amino acids) identity only. The GTGV/GT11.18 was also distantly related to the Southern screamer gyrovirus (MH016740.1) from the US in 2007^11^ with 52.4% identity only in the VP1 protein sequence.

The VP1 protein of the GTGV/GT11.18 contained the motif ^141^WWRWA^145^, and also had a high number of basic amino acids, especially arginines in its N-terminal region. The minimal signature motif for the protein tyrosine phosphatases (PTPase) superfamily CX5R (^46^CSCGNFR^52^) and the motif ^34^WX7HX3CXCX5H^54^ was found in the VP2 protein, suggesting that this protein may exhibit PTPase function^12,13^. It is also to be noted that the VP3 protein did not have any apoptin conserved protein domains.

### RNA viruses characterised from the duck samples

The RNA viruses, in addition to the picornaviruses already described^6^, detected and characterised from the duck samples belonged to the virus families *Reoviridae, Astroviridae, Caliciviridae* and *Coronaviridae.*

#### Avian orthoreovirus from Pacific black duck (PBDORV/PBD08.18)

**Figure 3:**
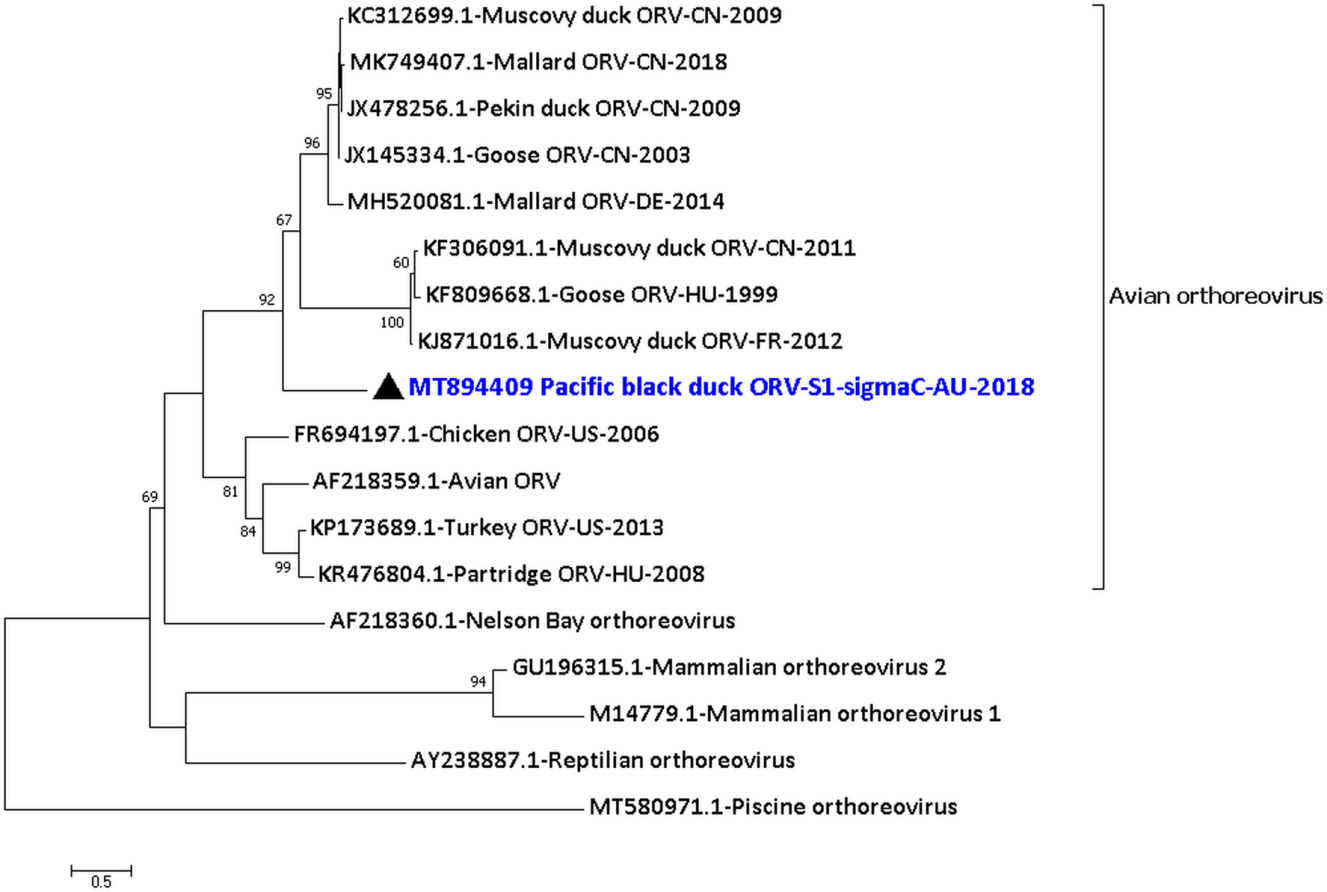
Phylogenetic analysis of the amino acid sequences of the sigma C protein of Pacific black duck orthoreovirus segment S1 (PBDORV/S1/PBD08.18). The amino acid sequences were aligned and analysed by using the maximum likelihood method based on the WAG + G + F model^73^ in MEGACC^74^ with a bootstrapping of 1000 replicates. The analysis involved 18 amino acid sequences and all positions containing gaps and missing data were eliminated. The final dataset contained a total of 257 amino acid positions. The numbers at the nodes represent bootstrap values and only bootstrap values at or above 60% are shown. Pacific black orthoreovirus is shown in blue and marked with a black triangle. No orthologous sigma C protein was found in Broome ORV, Baboon ORV and Mahlapitsi ORV.

From the Pacific black duck 08.18 sample, an orthoreovirus, family *Reoviridae*, was detected and characterised. All the 10 segments were detected and a total of 21,170 nucleotides (out of ~ 23 kb) of the consensus sequence of the Pacific black duck orthoreovirus genome were generated with a coverage from 2 to 336 (Supplementary material 1 Row 16–29). Table 2 provides the details on the avian orthoreovirus from the PBD08.18 sample along with the percentage identity to the closest relative of the nucleotide sequences and the proteins encoded by each segment. The analysis of the protein sequences showed that they were closely related to other avian orthoreoviruses with > 96% identity (for example, Fig. S2 of Supplementary material 2), except for the proteins p10, p17 and sigma C encoded by segment S1. However, the nucleotide sequences of the 10 segments of the PBDORV/PBD08.18 were about 84–96% identity to its closest relative, except for segment S1 (Table 2). The phylogenetic analysis of the assembled consensus sequence of the sigma C protein from segment S1 was most identical, but still distant, to the JX145334.1 Goose orthoreovirus sigma C protein from China in the year 2003^14^ and JX478256.1 Pekin duck orthoreovirus sigma C protein from China in the year 2009^15^ with 54.8% identity (187/341 amino acids) than to any other orthoreovirus sigma C proteins (Fig. 3). The partial p10 protein and complete p17 protein were most identical but still distant to MH520081.1 Mallard orthoreovirus p10 protein from Germany^16^ with 67.2% identity and to JX478256.1 Pekin duck orthoreovirus p17 protein from China^15^ with 52.1% identity, respectively (Table 2).

**Table 2:** Details on the avian orthoreovirus from Pacific black duck 08.18 sample (PBDORV/PBD08.18).

Segment|protein encoded	Length of the generated nucleotide sequence (nt)	Protein function	Percentage identity to its closest relative using MEGA	
			Amino acid	Nucleotide
L1|partial lambda (λ) A	3860	Core shell scaffold^42^	KC312700.1 Muscovy duck ORV^75^ with 99.0% identity	MH520075.1 Mallard duck ORV^16^ with 91.9% identity
L2|partial lambda (λ) B	3661	Putative transcriptase^42^	MH520077.1 Mallard ORV^16^ with 98.6% identity	MH520077.1 Mallard ORV^16^ with 93.1% identity
L3|partial lambda (λ) C	3624	Capping enzyme^42^	MK955820.1 Cherry valley duck ORV^14^ with 97.3% identity	JX145330.1 Goose ORV^14^ with 92.8% identity
M1|partial mu (µ) A	Two consensus sequences of 939 and 1199nt long	Putative transcriptase co-factor^42^	MH520078.1 Mallard ORV^16^ with 97.4% and 96.7% identity, respectively	KF306085.1 Muscovy duck ORV^76^ with 90.9% and 91.9% identity, respectively
M2|partial mu (µ) B	Two consensus sequences of 497 and 1066nt long	Part of the outer capsid and may help in the penetration to the host cell^42^	KR476802.1 Partridge ORV^77^ with 97.5% and 99.1% identity, respectively	KR476802.1 Partridge ORV^77^ with 84.1% and 85.8% identity, respectively
M3|partial mu (µ) NS	Two consensus sequences of 983 and 542nt long	Formation of virus factories and protein recruitment^42^	MH520080.1 Mallard ORV^16^ with 96.6% and 98.1% identity respectively	MH520080.1 Mallard ORV^16^ with 90.9% and 91.6% identity respectively
S1|partial p10, complete p17 and sigma (σ) C	Two consensus sequences of 211 and 1328nt long	σC forms part of the outer capsid that helps in host cell attachment of the virus particle^42^	p10: MH520081.1 Mallard ORV^16^ with 67.2% identity p17: JX478256.1 Pekin duck ORV^15^ with 52.1% identity σC: JX145334.1 Goose ORV^14^ and JX478256.1 Pekin duck ORV^15^ with 54.8% identity	1328nt sequence: JX478256.1 Pekin duck ORV^15^ and KC312699.1 Muscovy duck ORV with 67.6% identity
S2|sigma (σ) A	1269	Part of the inner core of the capsid^42^	KC508653.1 Muscovy duck ORV^78^ with 97.8% identity	KF306088.1 Muscovy duck ORV^76^ with 91.1% identity
S3|partial sigma (σ) B	1107	Part of the outer capsid^42^	MH520083.1 Mallard ORV^16^ with 96.6% identity	JX145336.1 Goose ORV^14^ with 85.5% identity
S4|partial sigma (σ) NS	884	ssRNA binding^42^	KJ871025.1 Muscovy duck ORV with 99.6% identity	KJ871015.1Muscovy duck ORV with 95.7% identity

#### Rotavirus G from Pacific black duck (PBDRVG/PBD08.18)

Rotavirus G also belongs to the virus family *Reoviridae* and was detected and characterised from the PBD08.18 sample*.* All the 11 segments of the virus were detected and characterised. A total of 17,808 nucleotides (out of ~ 18 kb) of the consensus sequence of the Pacific black duck rotavirus G genome were generated with a coverage of 2–512 (Supplementary material 1 Row 30–40). It should be noted that all the segments have been named as per the rotavirus G NCBI RefSeq sequence (NC021580.1 to NC021590.1). Table 3 provides the details on the PBDRVG/PBD08.18 along with the percentage identity to the closest relative for each segment. Out of the 11 proteins encoded by the 11 segments of Pacific black duck rotavirus G, seven proteins were most closely related to their orthologous proteins of Grey teal rotavirus G from south west Victoria, Australia from 2017^5^ with 87.2–99.6% identity (Table 3). Segment 7, encoding the NSP3 protein, was most closely related to the MK204407.1 Grey teal partial NSP3 protein (only 152 amino acids given) from Australia in the year 2017^5^ with 64.9% identity. The complete NSP3 protein of the PBDRVG/PBD08.18 was most identical but still distant to KY689682.1 Turkey rotavirus G from the US in the year 2016^17^ with 44.3% identity only. It should also be noted that the NSP4 protein was not generated from the Grey teal sample from Australia^5^ and hence was not available for comparison. The VP4 and the VP7 protein of the Pacific black duck rotavirus G were also distantly related to the orthologous proteins of Grey teal rotavirus G from Australia^5^. The nucleotide sequences of all the segments of the PBDRVG/PBD08.18 were only about 56–98% identity to its closest relative.

**Table 3:** Details on the rotavirus G from Pacific black duck 08.18 sample (PBDRVG/PBD08.18).

Segment|protein encoded	Length of the generated nucleotide sequence (nt)	Protein function	Percentage identity to its closest relative using MEGA	
			Amino acid	Nucleotide
S1|partial VP1	3479	RNA polymerase^45,46^	MK204401.1 Grey teal RVG^5^ with 98.6% identity	MK204401.1 Grey teal RVG^5^ with 92.6% identity
S2|partial VP2	2832	Part of the inner layer of the capsid and is associated with the RNA polymerase and the VP3^18^	MK204402.1 Grey teal RVG VP2^5^ with 98.5% identity	MK204402.1 Grey teal RVG^5^ with 79.8% identity
S3|partial VP4	2322	Part of the outer capsid^18^	KY689679.1 Turkey RVG^17^ with 54.3% identity	KY689679.1 Turkey RVG^17^ with 60.3% identity
S4|partial VP3	2277	A multifunctional protein which may be associated with guanylyltransferase^79^	MK204403.1 Grey teal RVG^5^ with 98.2% identity	MK204403.1 Grey teal RVG^5^ with 95.1% identity
S5|partial NSP1-1 and complete NSP1	1198	Innate host immune response regulator^80,81^	NSP-1 protein: MK204405.1 Grey teal RVG^5^ with 98.0% identity	MK204405.1 Grey teal RVG^5^ with 96.6% identity
S6|VP6	1272	Part of the intermediate layer of the capsid^44^	MK204406.1 Grey teal RVG^5^ with 87.2% identity	MK204406.1 Grey teal RVG^5^ with 73.8% identity
S7|NSP3	1276	Regulates the virus mRNA translation^18^	MK204407.1 Grey teal RVG partial NSP3 protein^5^ with 88.1% identity; KY689682.1 Turkey RVG^17^ with 60.4% identity	MK204407.1 Grey teal RVG partial S7^5^ with 89.1% identity; KY689682.1 Turkey RVG^17^ with 70.4% identity
S8|NSP2	997	Aids in the virus particle assembly^82^	MK204408.1 Grey teal RVG^5^ with 99.6% identity	MK204408.1 Grey teal RVG^5^ with 97.8% identity
S9|partial VP7	745	Part of the outer capsid^18^	MT025062.1 Gentoo penguin RVG with 66.7% identity and MK204409.1 Grey teal RVG with 66.4% identity	MT025062.1 Gentoo penguin RVG with 58.6% identity and MK204409.1 Grey teal RVG with 56.5% identity
S10|partial NSP4	772	Acts as viral enterotoxin^83^	MH453872.1 Ruddy turnstone RVG^19^ with 53.7% identity	MH453872.1 Ruddy turnstone RVG^19^ with 67.8% identity
S11|NSP5	613	Aids in the virus particle assembly^82^	MK204410.1 Grey teal RVG NSP5^5^ with 92.9% identity	MK204410.1 Grey teal RVG^5^ with 80.0% identity

**Figure 4:**
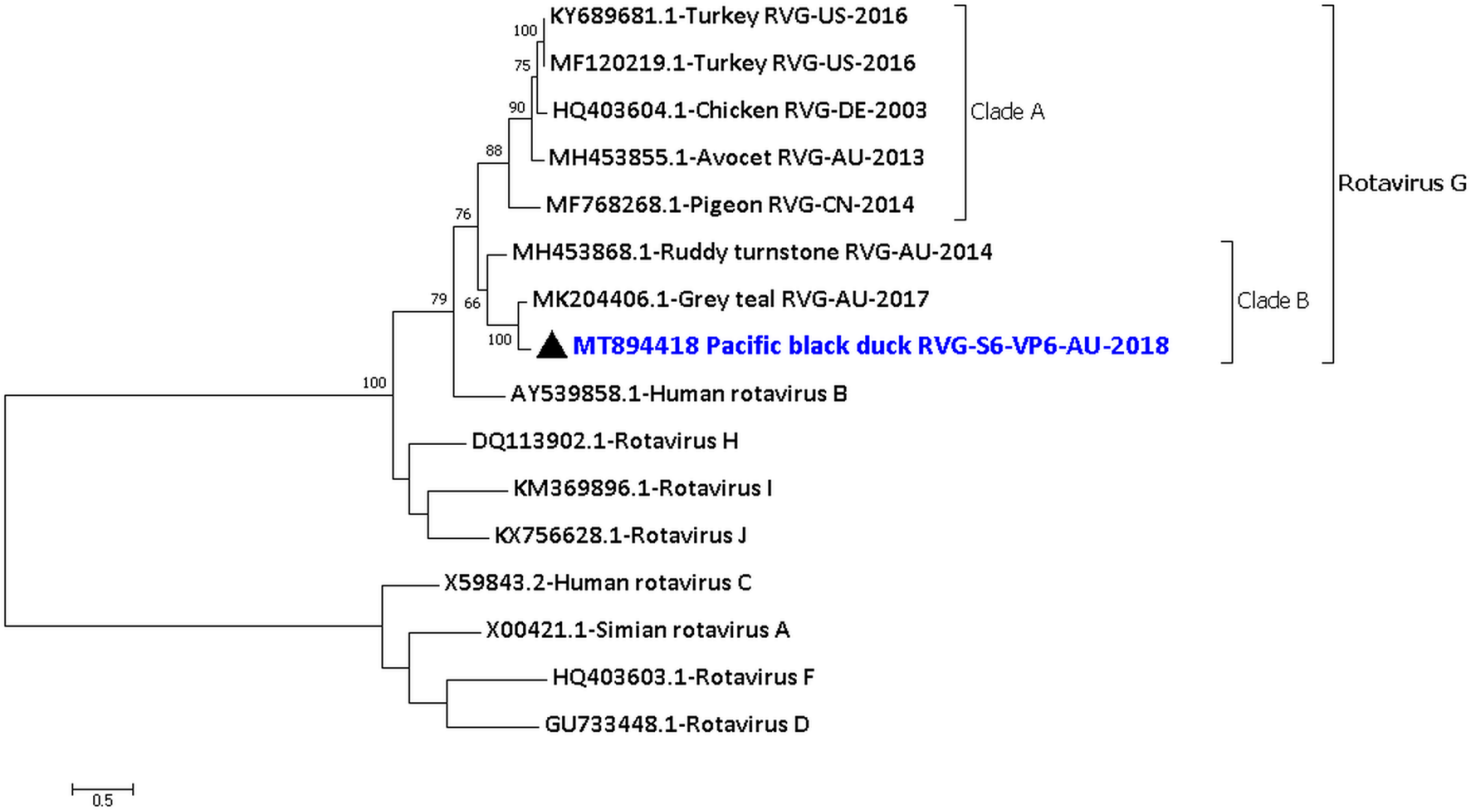
Phylogenetic analysis of the amino acid sequences of the VP6 protein of Pacific black duck rotavirus G segment S6 (PBDRVG/PBD08.18). The amino acid sequences were aligned and analysed by using the maximum likelihood method based on the LG + G + F model^72^ in MEGACC^74^ with a bootstrapping of 1000 replicates. The analysis involved 16 amino acid sequences and all positions containing gaps and missing data were eliminated. The final dataset contained a total of 371 amino acid positions. The numbers at the nodes represent bootstrap values and only bootstrap values at or above 60% are shown. Pacific black rotavirus G is shown in blue and marked with a black triangle.

From the phylogenetic analysis of the VP1 and the VP6 protein (Fig. 4 and Fig. S3 of Supplementary Material 2), it is clear that the rotavirus G forms two different clades. Clade A includes the rotavirus G from chicken^18^, turkey^17^, avocet^19^ and pigeon while Clade B includes the rotavirus G from the Pacific black duck, Grey teal^5^ and Ruddy turnstone^19^.

Various motifs found in the PBDRVG/PBD08.18 partial VP3 protein of segment 4 varied to those found in VP3 protein of the other rotavirus G. For example, in lieu of an EXXFXXXN motif in the N-terminal region^18^, the PBDRVG/PBD08.18 has an EXXFXXXS motif; and in lieu of an ALYXLSNXXN motif in the central region^18^, the PBDRVG/PBD08.18 has an ALYXISNXXN motif in the central region. Also, the putative guanylyl transferase KXTAMDXEXP and KXXGNNH motifs found in rotavirus A and C^20^ were not found in the Pacific black duck rotavirus G, as observed for other rotavirus G sequences^18^. The conserved domain HGXGHXRXV at 229 to 237 amino acid and histidine triad His-X-His-X-His-XX at 229–234 amino acid of the NSP2 protein that aid in the binding of nucleoside triphosphate^21^ was found in the NSP2 protein. The five fully conserved cysteine residues were found in the partial VP7 of the PBDRVG/PBD08.18 at the expected location, which in turns forms the disulphide bonds that is thought to stabilise the protein structure^18^.

#### Rotavirus F from Chestnut teal (CTRVF/CT08.18)

From the Chestnut teal 08.18 sample, a few good quality NGS reads of Chestnut teal rotavirus F were detected and characterised. The NGS reads were mapped to segment 7 of the virus encoding partial NSP3 protein. Further analysis led to the generation of a 647 nucleotides long consensus sequences with a coverage of 2–11 with a minimum mapping quality of 80 or higher (Supplementary material 1 Row 42). The partial NSP3 protein, which regulates the virus mRNA translation^18^, was relatively closely identical to the JQ920001.1 Chicken rotavirus F from Germany in the year 2003^18^ with 88.8% (191/215 amino acids) identity.

#### Avastrovirus from Pacific black duck (PBDAstV/PBD05.18) and Chestnut teal (CTAstV/CT11.18)

**Figure 5:**
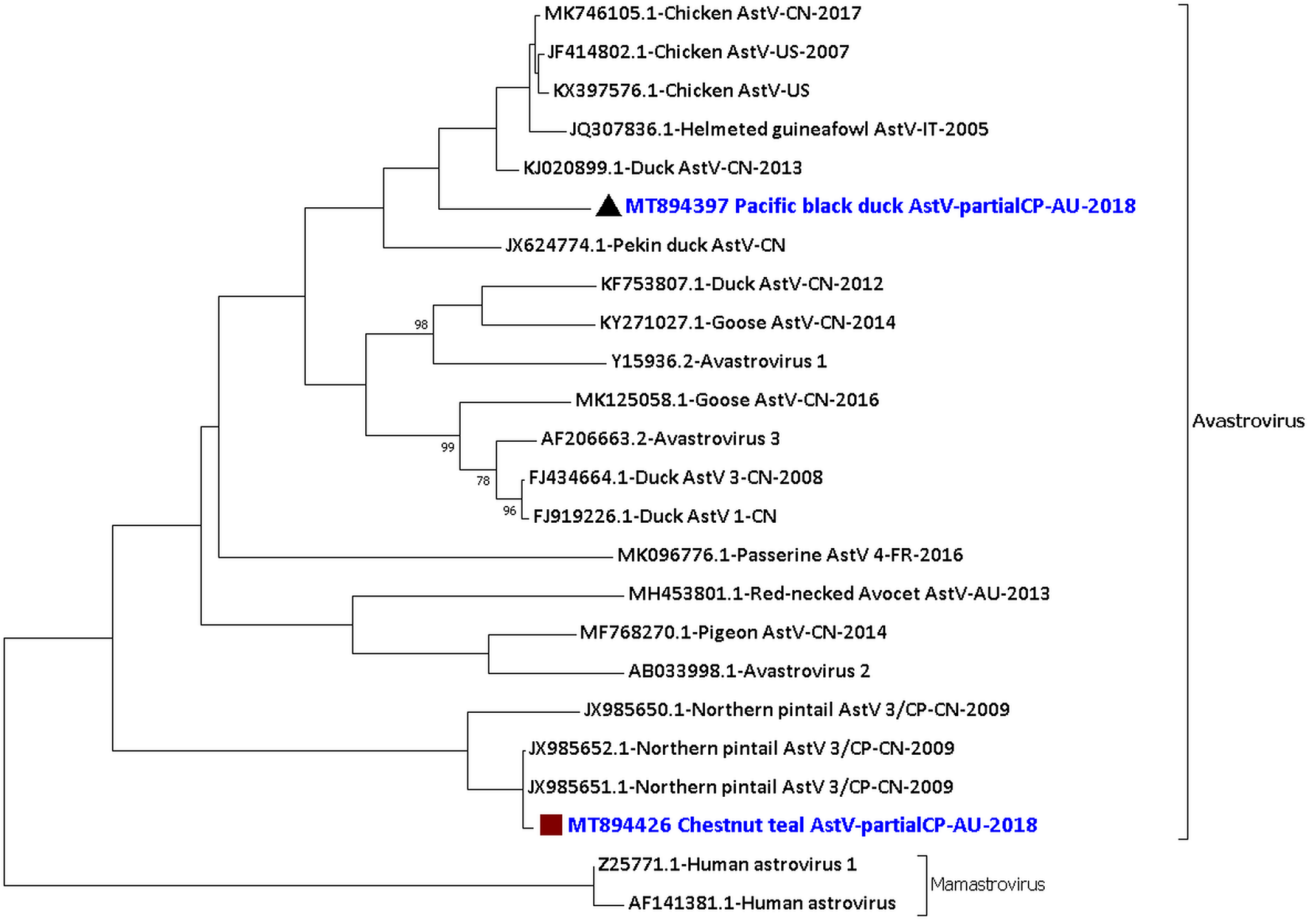
Phylogenetic analysis of the amino acid sequences of the partial capsid protein (ORF2) of Pacific black duck avastrovirus (PBDAstV/1297nt/PBD05.18) and Chestnut teal avastrovirus (CTAstV/1564nt/CT11.18). The amino acid sequences were aligned and analysed by using the maximum likelihood method based on the LG + G model^72^ in MEGACC^74^ with a bootstrapping of 1000 replicates. The analysis involved 24 amino acid sequences and all positions containing gaps and missing data were eliminated. The final dataset contained a total of 224 amino acid positions. The numbers at the nodes represent bootstrap values and only bootstrap values at or above 60% are shown. Pacific black avastrovirus is shown in blue and marked with a black triangle and Chestnut teal avastrovirus is shown in blue and marked with a brown square.

From the Pacific black duck 05.18 sample and Chestnut teal 11.18 sample we detected and characterised two astroviruses that belong to the virus family *Astroviridae* and the genus *Avastrovirus*. A total of 3443 nucleotides (out of ~ 7 kb) of the consensus sequence of the Pacific black duck avastrovirus genome was generated. In comparison, a total of 4185 nucleotides of the consensus sequence for the Chestnut teal avastrovirus genome was generated (Supplementary material 1 Row 12–15 and 43–46 and Supplementary material 2 Fig. S4). The -1 ribosomal frameshift signal was found to be from the 79th to 85th nucleotides of the 738 nucleotides long consensus sequence encoding partial ORF1a and ORF1b of the PBDAstV/PBD05.18 and from the 1026th to 1032nd nucleotides of the 1,554 nucleotides long consensus sequence encoding partial ORF1a and ORF1b of the CTAstV/CT11.18. The phylogenetic analysis of the partial capsid protein (ORF2) encoding consensus sequence (PBDAstV/1297nt/PBD05.18) of the PBDAstV showed that it was most identical, but still distant to KJ020899.1 Duck astrovirus from China in 2013^22^ with 83.1% (356/428 amino acids) identity only (Fig. 5). The phylogenetic analysis of the partial capsid protein (ORF2) encoding consensus sequence (CTAstV/1564nt/CT11.18) of the CTAstV showed that it was closely related to JX985715.1 Northern pintail astrovirus from China in the year 2009^23^ with 98.0% (251/256 amino acids) identity (Fig. 5) while having only 85.2% identity (655/768 nucleotides) at the nucleotide level. The amino acid sequence of the partial capsid region of Chestnut teal astrovirus (CTAstV/1564nt/CT11.18) is only 41.4% identical to the Pacific black duck astrovirus partial capsid region (PBDAstV/1297nt/PBD05.18). Analysis of other proteins encoded by the consensus sequences of the duck avastroviruses showed differences in the identity to their closest relative (Supplementary material 1 Column J).

#### Calicivirus from Pacific black duck (PBDCV/PBD12.16)

From the Pacific black duck December 2016 sample, we detected and characterised a duck calicivirus, belonging to the virus family *Caliciviridae*. A total of 4,710 nucleotides (out of ~ 8.3 kb) of the consensus sequence of the virus genome were generated. Five consensus sequences of 305 nucleotides, 1596 nucleotides, 331 nucleotides, 1129 nucleotides, and 1112 nucleotides encoding partial ORF1 were generated with a coverage from 2–90 (Supplementary material 1 Row 6–10). Another consensus sequence of 237 nucleotides long was generated encoding partial VP2 protein (Supplementary material 1 Row 11). These sequences had the characteristic motifs of a calicivirus at the expected location in the amino acid sequence (Supplementary material 1 Column K). The Pacific black duck calicivirus consensus sequences showed 96.3% to 97.2% identity at the nucleotide level (blastn) to the MK204392.1 Grey teal calicivirus from south west Victoria, Australia in the year 2017^5^. Phylogenetic analysis of the amino acid sequence of the partial ORF1 containing Helicase motif (PBDCV/1596nt/PBD12.16) and the RdRp region (PBDCV/1129nt/PBD12.16) showed these to be 100% identical to the MK204392.1 Grey teal calicivirus ORF1 protein. In comparison, the VP1 region (PBDCV/1112nt/PBD12.16) was 99.4% (368/370 amino acids) identical to the MK204392.1 Grey teal calicivirus ORF1 protein (Fig. S5 of Supplementary material 2), while being distantly related to the other caliciviruses with 36.2–45.6% (134–169/370 amino acid) identity only. However, based on the phylogenetic analysis of the VP1 region and genus demarcation criteria of the family *Caliciviridae*^24^, the calicivirus from the PBD12.16 sample belongs to the genus *Nacovirus*.

#### Gammacoronavirus from Chestnut teal

From the Chestnut teal 08.18 sample, a few good quality reads of gammacoronavirus were detected and characterised. Further analysis led to the generation of a short 382 nucleotides long consensus sequence with a coverage of 2–9 (Supplementary material 1 Row 41). The BLASTN analysis of the short consensus sequences shows that it is 100% identical to the MK204393.1 Grey teal gammacoronavirus from Australia in the year 2017^5^. Further analysis of this virus was not carried out due to the low quantity and short consensus sequence.

## Discussion

Previously, we described in detail the metagenomics methodology that can be used to detect and characterise both DNA and RNA viruses from bird faecal samples^3^. Using this method, we have now conducted a surveillance study of Australian wild ducks from which faecal samples were collected at different time points from a single location. As reported earlier^1,3,4,6^ and also shown in the current study, we were able to detect and characterise both DNA and RNA viruses. We were also able to characterise novel viruses from these ducks^6^.

In the current study, details on the genome sequences of a duck aviadenovirus genetically different to currently known waterfowl aviadenoviruses and a novel gyrovirus are provided. We also report what, to the best of our knowledge, is the first finding of an avian orthoreovirus from Pacific black ducks and a rotavirus F from Chestnut teals, albeit being a short partial sequence from segment 7 of the rotavirus genome.

The Pacific black duck aviadenovirus characterised here belongs to the genus *Aviadenovirus*. Adenoviruses belonging to the genus *Aviadenovirus* have been found in, but not limited to, chickens^25^, turkeys^26^, geese^8^, pigeons and ducks^7^. The abundance of the Pacific black duck aviadenovirus in the juvenile Pacific black ducks faecal sample (PBD12.16) might indicate active infection and shedding of the virus from these birds. Analysis of the virus proteins showed that some of them were more identical but still distant to their counterparts expressed by the KJ469653.1 Muscovy duck adenovirus 2, while others to that of JF510462.1 Goose adenovirus 4. Nevertheless, the Pacific black duck aviadenovirus was almost equidistant from both the viruses in the maximum likelihood tree generated. This analysis is consistent with our previous analysis of the PBDAdV/PBD12.16 virus, as reported from the very short sequences generated^3^ before resequencing of the sample. From the central region of the partial Pacific black duck aviadenovirus genome, 18 genes (IVa2–fibre) were found in total which are common to all adenoviruses and said to be inherited from a common ancestor of all known adenoviruses^8^. However, only a partial fibre gene sequence was generated from the NGS data, and hence at this point, it is uncertain if the PBDAdV/PBD12.16 have one fibre gene like the Fowl adenovirus 8^25^ or two fibre genes like the Goose adenovirus 4^8^. Among the ORFs, ORF2 contained Parvovirus NS1 superfamily domain at 2nd–191st amino acids, as seen in some of the other waterfowl aviadenoviruses^8^. As reported earlier^6^, in the PBD12.16 sample, we detected an adeno-associated virus which may utilise the Pacific black duck aviadenovirus as a helper virus for its replication. However, currently, how the ORF2 protein assists the adeno-associated virus replication is uncertain.

A novel gyrovirus was found in the Grey teal 11.18 sample and was distantly related to other gyroviruses. Gyroviruses have been recently reclassified in the *Anelloviridae* family^12^. Gyroviruses have been found in cloacal, oral, and blood samples from birds^27–29^ and faecal samples of carnivorous mammals such as ferrets, cats and also humans^30–34^. However, the natural host of gyroviruses is thought to be birds. The finding of the GTGV/GT11.18 contributes to the wide genetic diversity of the gyroviruses. The Southern screamer gyrovirus (MH016740.1)^11^, the only other gyrovirus from a bird belonging to the order *Anseriformes* yet characterised, was distantly related to the Grey teal gyrovirus. Despite being very different to currently known gyroviruses, genetic analysis revealed that the N-terminal region of the VP1 protein is rich in basic amino acids and is a characteristic shared by all members of the *Anelloviridae* family^12^. The motif ^173^WWRWA^177^ in the VP1 protein of the GTGV/GT11.18 also appears to be conserved for all gyroviruses^30^. The VP2 of the GTGV/GT11.18 has the characteristic motif of protein tyrosine phosphatases which is the CX5R motif and the WX7HX3CXCX5H motif, that is conserved in other gyroviruses and anelloviruses^12,13^. A minimal signature motif with the configuration CX5R is highly conserved in all PTPases. PTPases catalyse the removal of phosphate from phosphotyrosine^35^ and may be important for the replication of the virus^13^. The VP3 protein of many gyroviruses, such as chicken anaemia virus, has some important motifs related to the induction of apoptosis in infected cells^29,36–38^. However, similar to some other gyroviruses from zoo and wild birds, no such related motif was found in the GTGV/GT11.18.

Reoviruses were detected and characterised in both Pacific black ducks and Chestnut teals collected in August 2018, which suggest that these reovirus infections may be more common in winter compared to other seasons. Other reovirus seasonal distribution studies in humans and chickens also show reovirus infections to be high during colder months of the year^39–41^.

The sigma C protein of the Pacific black duck orthoreovirus that forms part of the outer capsid and helps in host cell attachment of the virus particle^42^ was only 54.5% identical to its nearest homologous avian virus protein counterpart. This may be because of a specific host adaptation and to the best of our knowledge, this is the first report of an avian orthoreovirus from Pacific black ducks and more widely from a wild duck in Australia. It is also to be noted that sigma C is the most variable protein of avian orthoreoviruses and antibodies to this protein can neutralise the virus infection^43^. Nevertheless, further studies are required to determine whether the Pacific black duck orthoreovirus can infect other closely related host species that can lead to reassortment. However, the analysis of the other encoded proteins of the virus suggests that this virus may infect closely related host species leading to reassortment.

All the 11 segments of a Pacific black duck rotavirus G were detected and characterised from the PBD08.18 sample. The phylogenetic analysis of the VP6 protein, one of the most conserved proteins that form the intermediate layer of the capsid^44^, and the phylogenetic analysis of the VP1 protein that functions as the RdRp^45,46^ shows that rotavirus G forms two different clades. Clade B includes the rotavirus G from the Pacific black duck, Grey teal^5^ and Ruddy turnstone^19^ (all three viruses from Australia). In contrast, clade A includes the rotavirus G from chicken^18^, turkey^17^, avocet^19^ (from Australia) and pigeon (unpublished). The most diverse segments of PBDRVG/PBD08.18 were S3 (VP4 protein), S9 (VP7 protein) and S10 (NSP4 protein). VP4 and VP7 form part of the outer capsid and the genetic diversity may be due to host-related immune selection pressure. However, the NSP4, which act as a viral enterotoxin, is only 53.7% identical to its closest relative and could indicate the presence of a diverse group of rotavirus G in this wildlife population that may exhibit different patterns of virulence in these hosts.

The complete genome of rotavirus F has only been molecularly characterised from chickens^18^, although partial sequences of the same have been detected and characterised from a pig (unpublished) and partridge^47^. The current study is the first to detect and characterise rotavirus F from a duck species, albeit only a partial segment being identified. This is due to the low amount of virus (0.0001% of NGS reads) present in the sample. The genetic diversity and evolution of rotavirus F are less understood due to the limited characterisation of the virus, and further studies involving more infected duck samples may help in its elucidation.

The phylogenetic analysis of the avastrovirus from Pacific black ducks and Chestnut teals suggest that these viruses are quite different to one another with ~ 60% difference albeit being detected from the same location at different time points. This indicates the wide genetic diversity of the avastroviruses.

The Pacific black duck orthoreovirus (except for the segment S1) and the Pacific black duck rotavirus G from the PBD08.18 sample, and the Chestnut teal avastrovirus from the CT11.18 sample showed conservation at the amino acid sequences while being more distantly related at the nucleotide level to their closest relatives, respectively. Taken together, this may indicate that there is significant evolutionary distance/time between a common ancestor of the viruses, but that conservation at the amino acid sequence of the protein is subjected to a strong selective pressure. The conservation of protein structure across different avian hosts may also indicate that these viruses may be capable of cross-species infection or that cross-species infection is part of their evolutionary history. The close relatedness of the Northern pintail avastrovirus detected from China^23^ to the Chestnut teal avastrovirus suggests that the migration of birds (likely migratory shorebirds) along the East Asian—Australasian Flyway could be important in the evolution of these viruses, as similarly observed for an avian paramyxovirus 6 characterised earlier from a Pacific black duck sample collected from the same site 18 months prior to this sample^3^.

The Pacific black duck calicivirus is very closely related to the MK204392.1 Grey teal calicivirus from south west Victoria, Australia^5^. The Pacific black duck collection site is about 375 km away from the MK204392.1 Grey teal calicivirus collection site and was taken three months prior to the sample collected from the Grey teal. Both Pacific black duck and Grey teal are dabbling ducks found in Australia that may share habitat at any particular time point. Dabbling ducks can travel about 25 km to hundreds of kilometres per day^48,49^. Caliciviruses are thought to be transmitted through direct contact with an infected host or indirectly via oral-faecal route^50^. Thus, the calicivirus detected in the current study may indicate cross-species transmission and infection occurs between these related duck species.

It should be noted that at the collection time, no clinical signs of disease were observed in the ducks and no close physical or extended examination such as post mortem or histopathological examination, was carried out. However, the high abundance of virus reads for some of the viruses from the bird samples may indicate an active infection and shedding of the virus. Aviadenoviruses can cause gizzard erosion or severe, often fatal cases of hepatitis-hydropericardium syndrome or inclusion body hepatitis depending on the virus and the host species^25,51,52^. For viruses in the genus *Gyrovirus*, Chicken anaemia virus is so far known to be the only pathogenic member of that genus^12^ causing an economically important clinical and subclinical disease in young chickens, with a worldwide distribution^53^. Nonetheless, a gyrovirus was also sequenced from Northern fulmar (*Fulmarus glacialis*) and Southern screamers (*Chauna torquata*) with neurological clinical signs^11,28^, as well as from healthy birds^11^.

Although avian orthoreoviruses have been associated with various disease conditions, a direct link between the virus and disease has been demonstrated only for viral arthritis syndrome or tenosynovitis in chickens^42^. However, avian orthoreoviruses were sequenced from diseased Muscovy ducks that had severe haemorrhagic and necrotic lesions in the liver and spleen^54^ and from Pekin ducks that had spleen necrosis^55^. Rotavirus G and rotavirus F were found in chickens with the runting-stunting syndrome; however, its role in the disease remains unclear^56^.

Among birds, astroviruses have been associated with various diseases such as enteritis, hepatitis, nephritis and runting-stunning syndrome^57–59^. However, the pathogenicity of the viruses described in the current study is currently unknown, and further studies involving histopathological examinations are required for its elucidation. Nevertheless, all of the viruses described in the current study may spread through the faecal-oral route^27,42,60^ and may cause asymptomatic infections as observed of the wild ducks in the current study.

The current study was carried out by sampling several representative bird species from a bird community at specific time points from a single location in an effort to comprehend virus diversity, abundance and seasonal prevalence. The Grey teal gyrovirus was the most abundant virus (414 reads at Q ≥ 20) from the GT11.18 sample and exceeded the two parvoviruses previously reported from this sample with 141 and 222 reads at Q ≥ 20 respectively^6^. The generation of the partial sequences of the other avian viruses from the duck samples described here may be due to various factors, including but not limited to, virus particle quantity, stage of infection and library preparation. Also, as stated earlier, the most abundant avian virus from each of the duck samples, except for the GT11.18 sample, was either an avian parvovirus or an avian picornavirus^6^. Future studies involving more extensive sample collection may lead to the complete genome elucidation of the viruses described here. However, the presence of motifs at the expected location in the generated virus consensus sequences suggests that they form part of a functional virus genome, although the description of each motif functions is beyond the scope of the manuscript. Furthermore, taken together with the previously described viruses^1,3,4,6^, the detection of more than one virus from each sample indicates co-infection, which could have a more significant health impact on the host when compared to infection with an individual virus.

Detection and characterisation of several potentially pathogenic avian viruses, some genetically novel while others similar to those detected in other avian hosts, indicates further virus surveillance studies of wild ducks are warranted. Ducks being a natural reservoir of pathogenic and zoonotic viruses should be monitored continuously to detect any potential threat to other wildlife and humans. Future pathological studies on the pathogenicity of the characterised viruses will also aid in comprehending the host–virus relationship.

## Materials and methods

### Sample collection

The current study uses the samples reported earlier^6^. Fresh wild duck faecal samples of Pacific black ducks (PBD), Chestnut teals (CT), Grey teals (GT) and Wood ducks (WD) were collected in May 2018 (late autumn), August 2018 (late winter), November 2018 and December 2016 (late spring/early summer) from Wallington, south-eastern Victoria, Australia. Pacific black ducks were captured in May 2018 (PBD05.18), August 2018 (PBD08.18) and December 2016 (PBD12.16 (described as MAD previously^3^)). Chestnut teal samples were collected in May (CT05.18), August (CT08.18) and November 2018 (CT11.18). Grey teal samples were collected in November 2018 (GT11.18) only, while Wood duck samples were collected in August 2018 (WD08.18) only. All samples were stored at − 80 °C within 1–3 h of collection until processing.

Bird sample collection was approved under Deakin University’s Animal Ethics Committee project number B43–2016 and Department of Environment, Land, Water and Planning permit number 1008206. The current study involving these samples were performed in accordance with relevant guidelines and regulations.

### Virus enrichment from samples

Virus particles enrichment and virus nucleic acid extraction was carried out as per a previously optimised protocol in our laboratory^3^. Briefly, the faecal samples were subjected to various biophysical methods such as homogenisation (25 Hz for 2 min), centrifugation (17000×*g* for 3 min) and filtration using a 0.8 µm PES filter (17000×*g* for 1 min). The sample was then divided into two aliquots (aliquot A—ultracentrifuged, aliquot B—non-ultracentrifuged) as described earlier^3^. Both aliquots were then nuclease treated using benzonase and micrococcal nuclease (37 °C for 2 h), followed by nucleic acid extraction using the QIAamp Viral RNA mini kit (Qiagen), as previously described^3^.

### Next-generation sequencing (NGS)

cDNA synthesis and amplification was carried out using the SeqPlex RNA Amplification Kit (Sigma) as per the manufacturer’s instructions using the extracted virus nucleic acids from both aliquots of the samples. Next generation sequencing library preparation was performed using the Ion Fragment Library kit (Life Technologies) followed by quantification of libraries, as described earlier^3^. Libraries were then pooled and were loaded onto Ion 530 or 540 chips using the Ion Chef Instrument. Template preparation was carried out and then the chips were run on an Ion Torrent S5XL System (Thermo Fisher Scientific) as per company protocols and as described earlier^3^.

### NGS data analyses

NGS data analyses were carried out as described earlier^3,6,61,62^. Two local BLAST datasets were created for all the available virus sequences and all RefSeq virus sequences from the NCBI GenBank genetic sequence database (Dec 2018), as described earlier^6^. BLASTN and TBLASTX query against the two virus reference sequence databases was performed with an e-value cut-off of 1 × 10^−10^ and 1 × 10^–30^. The query results files were converted into spreadsheet files, sorted by virus matches, a list of potential virus targets created and then viruses of interest were identified for each sample, as described earlier^3,6^.

The NGS reads were mapped against reference virus genomes of interest using the TMAP plugin on the Ion Torrent server. The mapped reads were used to obtain full or partial consensus sequences of the viruses using Integrative Genomics Viewer software (IGV) (Broad Institute, MA, USA), as described earlier^3,6,61,62^. Contigs were generated from the AssemblerSPAdes 5.6.0 plugin on the Ion Torrent Server by using the NGS reads of each sample. The contigs were bioinformatically analysed to identify virus sequences as described above using the same e-value cut-offs. Virus contigs (> 500 nucleotides) were then used as references in TMAP plugin and trimmed to regions with a mapping quality of 80 or higher and a coverage depth of at least 2 unless specified, as described earlier^3,6^. Virus sequences were also generated by assembling overlapping sequences from contigs and consensus sequences by using MEGA7 and magicblast^63^. Final virus consensus sequences were then generated after subjecting the sequences again to TMAP and mapping using IGV, as described above.

### Grey teal gyrovirus PCR and Sanger sequencing

Real-time PCR amplification and Sanger sequencing were carried out for Grey teal gyrovirus from the GT11.18 sample for the region from 264th to 761st nucleotide, as a potential indication of this virus being different from other gyroviruses was observed during NGS analysis. The GTGV/GT11.18 was the most abundant virus from the sample compared to the two parvoviruses previously reported^6^, and the region of PCR analysis had NGS reads with very low abundance. Primer-BLAST^64^ software was used to design two sets of primers (Micormon—Monash University, Victoria, Australia). FP1-GTGV-347nt (5′-CCCGGAAACCTGTACGAGTC-3′) RP1-GTGV-761nt (5′-TCTCCTGTAGTGGCGTCTGA-3′) and FP2-GTGV-264nt (5′-TCCTGTGGCAACTTTCGGAG-3′) RP2-GTGV-756nt (5′-TGTAGTGGCGTCTGAATCGG-3′) were used as the forward and reverse primers. The PCR master mix was prepared with 2X AmpliTaq Gold™ 360 Master Mix with 1 mM of each primer and 2 µl of DNA in a total reaction volume of 10 µl. The PCR reaction was carried out in QuantStudio™ Flex 6 real-time thermal cycler (Applied Biosystems) using the following conditions: 95 °C for 10 min, 40 cycles of 95 °C for 30 s, 54 °C for 30 s, 72 °C for 30 s, followed by a final step of 72 °C for 3 min. This is followed by a melt curve stage of 95 °C for 15 s, 60 °C for 1 min and 95 °C for 15 s and a final hold at 4 °C. The two PCR products of ~ 434 bp size and ~ 512 bp size, respectively, were purified using the 2% Size Select E-Gel System (Thermo Fisher, USA). These products were sequenced using the Big Dye Terminator Cycle v3.1 on a Hitachi 3500XL Genetic Analyzer (Applied Biosystems, USA) as per the manufacture instructions.

### ORF prediction and motif analysis

Basic Local Alignment Search Tool^65,66^ (BLASTN, BLASTX and BLASTP), NCBI ORFfinder^67^, ScanProsite^68^ and PSORT II^69^ were used for identifying open reading frames (ORFs), and features of the virus genomes such as promotors, motifs and nuclear-locating signals, as described earlier^6^. The presence of these features in their expected location was used as an additional confirmation of the generated consensus sequences.

### Phylogenetic analysis of virus sequences

Relevant related sequences were selected from the NCBI GenBank database. Nucleotide and protein sequences were aligned using Clustal-W^70^ in MEGA 7^71^ software. MEGA7/MEGACC software was used to identify the best evolutionary model and the selected model was then used for generating the maximum likelihood tree with a bootstrapping of 1000 replicates. The distance data between sequences were calculated using the same software.

## Supplementary Information

Supplementary Information.

## Data Availability

All sequences analysed have been deposited in NCBI GenBank under accession numbers MT894381-MT894428. Other datasets generated or analysed during the current study are available from the corresponding author on reasonable request.
