# Supplementary material for: Metagenomic characterisation of additional and novel avian viruses from Australian wild ducks
Source: Sci Rep. 2020 Dec 17;10:22284. doi: 10.1038/s41598-020-79413-9 (PMC7747739; doi:10.1038/s41598-020-79413-9)
Supplement: Supplementary file 1 — Supplementary Information. [file 41598_2020_79413_MOESM1_ESM.pdf]

Supplementary material 1

Metagenomic characterisation of additional and novel avian viruses from Australian wild ducks

Running title: Additional Avian DNA and RNA viruses from Australian wild ducks

Supplementary material 1: Details of the sequences of the additional avian viruses found in the duck samples

Authors:
Jessy Vibin*[1,2], Anthony Chamings[1,2], Marcel Klaassen[3], Soren Alexandersen*[1,2,4]
1 Geelong Centre for Emerging Infectious Diseases, Geelong, VIC 3220, Australia;
2 School of Medicine, Deakin University, Geelong, VIC 3220, Australia;
3 Centre for Integrative Ecology, Deakin University, Waurn Ponds, VIC 3216, Australia;
4 Barwon Health, Geelong, VIC 3220, Australia;

*Corresponding Author: jessyatall@gmail.com, soren.alexandersen@deakin.edu.au

Supplementary material 1: Details of the sequences of the additional avian viruses found in the bird samples

The table provides details of the ORF of the sequences we provide. The first column gives the virus and the bird from which it was isolated. It also gives the NCBI accession number of the virus sequence. The 2nd column gives the coverage and mapping quality of the virus consensus sequence. The 3rd column provides the coverage analysis i.e the total number of reads generated for the particular virus sequences at a mapping quality on or above 20 with the minimum aligned length of 100. It also provides the abundance of the virus in the generated NGS data of the sample. The 4th column provides the protein the virus sequence encodes. The 5th – 9th column provides if the protein expressed in a sense or an antisense strand and the start of coding frame position, the nucleotide position of the start of the protein, the nucleotide position of the stop codon/end of the protein and the total length of the ORF. The 10th column gives the percentage of nucleotide/amino acid identity to its closest relative identified through either MEGA analysis or BLASTP. Finally, the last column if any features were identified in the sequene using ScanProsite, PSORT or GPMiner.

	Column A	B	C	D	E	F	G	H	I	J	K
	**Virus | Bird sample | NCBI Accession number**	**Coverage | Mapping quality (at which the final consensus sequences were generated)**	**Coverage analysis (Total no. of reads @ Q20) | Abundance (%)**	**ORF**	**Strand**	**Frame**	**Start**	**Stop**	**Length (nt | aa)**	**Nucleotide/amino acid identity to its closest relative (%)**	**Special features (Promoter / Motifs)**
	**Pacific black duck**										
	**PBD 12.16 sample**										
1	**Pacific black duck aviadenovirus** (PBDAdV)/11921nt | PBD12.16 | MT894381	2-1471 | Q80	15978 | 0.21%	dUTPase	+	2	668	1114	447 | 148	JF510462.1: 67.8% in aa (blastp)	ORF2 contained Parvovirus NS1 superfamily domain at 2-191 aa. ORF14 contains DEXH-box helicase domain of Mot1 at 82-196 aa. IVa2 contains AAA - ATPases at 98-141 aa. DNA polymerase type B family. N-terminal domain of the transcription elongation factor GreA at 502-556 aa, Phospholipase A2 aspartic acid active site at 1056-1066 aa, pat4 (RKKR) (5) at 13th aa, pat4 (RPKK) (4) at 20th aa and 2nd peroxisomal targeting signal (RIKKPNTQL) at 1036th aa of DNA polymerase ORF. Arginine-rich region profile at 297-350 aa and 2nd peroxisomal targeting signal (RIAADMGQL) at 139th aa of pTP. pat4 (RRRP) (4) at 18th aa of partial L1 52K.
				ORF52	+	2	1115	1726	612 | 203	KJ469653.1: 60.2% in aa (blastp)	
				ORF2	+	3	1908	2753	846 | 281	KJ469653.1: 47.5% in aa (blastp)	
				ORF14	-	3	3447	2779	669 | 222	KR135164.1: 47.6% in aa (blastp)	
				ORF12	-	1	4298	3507	792 | 263	KJ469653.1: 47.4% in aa (blastp)	
				IVa2	-	3	5559	4384	1176 | 391	JF510462.1: 80.3% in aa (blastp)	
				DNA polymerase	-	1	9329	5562	3768 | 1255	KJ469653.1: 81.9% in DNA polymerase aa (MEGA analysis)	
				pTP	-	2	11104	9332	1773 | 590	KJ469653.1: 69.1% in aa (blastp)	
				partial L1 52K	+	3	11187	>11921	735 | 244	KJ469653.1: 72.3% in aa (blastp)	

2	PBDAdV/14847nt | PBD12.16 | MT894382	5-4456 | Q80	36692 | 0.50%	partial L1 52K	+	2	<2	406	405 | 134	JF510462.1: 79.1% in aa (blastp)	Arginine-rich region profile at 18-83 aa, Bipartite nuclear localization signal at 28-42 aa, pat4 (RRRP) (4) at 27th aa and pat4 (RRRR) (5) at 78th aa of pVII. pat4 (KRRR) (5) at 12th aa, pat4 (RKRP) (4) at 29th aa, pat4: RRRR (5) at 107th aa, pat7 (PKSRKRP) (5) at 26th aa, bipartite NLS (RRSSSTIKLPKLPKSRK) at 14th aa of pX. Proline-rich region profile at 137-196 aa, pat4 (KRRR) (5) at 198th aa, pat7 (PPPAKRR) (3) at 194th aa of pVI. pat4 (KRKK) (5) at 25th aa of DNA binding protein. Transcription initiation factor IIF at 95-197 aa of 100K. Bipartite nuclear localization signal (RRIVPPTPQNSPKKSKK) at 68-84th aa, pat7 (PKKSKKM) (5) at 79th aa of 22K. pat4 (RRKP) (4) at 51th aa of U-exon. pat4 (RKRP) (4) at 3rd aa of partial fiber.
				Hexon-associated protein (IIIa)	+	3	390	2099	1710 | 569	KJ469653.1: 79.0% in aa (blastp)	
				penton base protein	+	3	2136	3668	1533 | 510	JF510462.1: 78.6% in aa (blastp)	
				pVII	+	3	3672	3923	252 | 83	KR135164.1: 74.6% in aa (blastp)	
				pX	+	3	3957	4505	549 | 182	KJ469653.1: 74.0% in aa (blastp)	
				Minor capsid protein VI	+	3	4551	5243	693 | 230	KR135164.1: 77.8% in aa (blastp)	
				Hexon	+	1	5284	8094	2811 | 936	JF510462.1: 80.7% in aa (blastp)	
				endoprotease	+	1	8113	8736	624 | 207	KR135164.1: 75.0% in aa (blastp)	
				DNA-binding protein	-	3	10024	8795	1230 | 409	KR135164.1: 69.3% in aa (blastp)	
				100K	+	2	10280	13024	2745 | 914	KJ469653.1: 59.0% in aa (blastp)	
				encapsidation protein 22K	+	3	12750	13256	507 | 168	KR135164.1: 43.2% in aa (blastp)	
				protein 33K	+	1	13168	13455	288 | 95	KR135164.1: 65.4% in aa (blastp)	
				hexon associated protein (protein VIII)	+	3	13479	14201	723 | 240	JF510462.1: 74.6% in aa (blastp)	
				U-exon	-	3	14425	14081	345 | 114	GU188428.1: 57.3% in aa (blastp)	
				partial fibre	+	1	14467	>14847	381 | 126	KJ469653.1: 44.4% in aa (blastp)	
3	PBDAdV/1891nt | PBD12.16 | MT894383	2-240 | Q80	750 | 0.01%	partial fibre	+	3	<3	1595	1593 | 530	KJ469653.1: 35.5% in aa (blastp)	
				partial ORF22	-	2	<1890	1609	282 | 93	KJ469653.1: 50.5% in aa (blastp)	
4	PBDAdV/4225nt | PBD12.16 | MT894384	2-318 | Q80	1193 | 0.01%	ORF20	-	1	1141	299	843 | 180	KJ469653.1: 52.9% in aa (blastp)	
				ORF56	-	2	1836	1426	411 | 136	JF510462.1: 45.2% in aa (blastp)	
				ORF19 (lipase)	-	1	3955	2231	1725 | 574	JF510462.1: 56.9% in aa (blastp)	
5	PBDAdV/450nt | PBD12.16 | MT894385	2-35 | Q80	49 | 0.0006%	partial ORF54	-	1	264	>1	300 | 99	JF510462.1: 51.0% in aa (blastp)	pat4 (RKPK) (4) at 19th aa of partial ORF19B.
				partial ORF19B	-	1	<450	301	150 | 49	JF510462.1: 70.0% in aa (blastp)	
6	PBDAdV/3188nt | PBD12.16 | MT894386	3-620 | Q80	1761 | 0.0240%	partial ORF19B	-	2	<3187	>2	3186 | 1061	JF510462.1: 71.7% in aa (blastp)	Lipase domain at 353-560 aa, Human PARM-1 domain at 963-1055 aa, Threonine-rich region at 928-1054 aa
7	PBDAdV/1310nt | PBD12.16 | MT894387	2-604 | Q80	1533 | 0.0209%	ORF53	-	2	823	239	585 | 194	JF510462.1: 39.3% in aa (blastp)	

6	**Pacific black duck calicivirus** (PBDCV)/ 305nt | PBD12.16 | MT894389	2-20 | Q80	27 | 0.0003%	partial ORF1	+	1	<1	>303	303 | 100	MK204392.1: 99.0% in aa (blastp)	
7	PBDCV/1596nt | PBD12.16 | MT894390	2-57 | Q80	176 | 0.0024%	partial ORF1	+	3	<3	>1595	1593 | 530	MK204392.1: 100% in aa (MEGA analysis)	Helicase at 33-193 aa
8	PBDCV/331nt | PBD12.16 | MT894391	2-8 | Q80	9 | 0.0001%	partial ORF1	+	3	<3	>329	327 | 108	MK204392.1: 99.0% in aa (blastp)	Caliciviridae 3C-like protein at 1-109 aa
9	PBDCV/1129nt | PBD12.16 | MT894392	2-80 | Q80	236 | 0.0035%	partial ORF1	+	3	<3	>1127	1125 | 374	MK204392.1: 100% in aa (MEGA analysis)	RdRp at 1-322 aa.
10	PBDCV/1112nt | PBD12.16 | MT894393	2-90 | Q80	156 | 0.0021%	partial ORF1	+	2	<2	>1111	1110 | 370	MK204392.1: 99.4% in aa (MEGA analysis)	Calicivirus coat protein at 3-206 aa
11	PBDCV/237nt | PBD12.16 | MT894394	2-38 | Q80	42 | 0.0005%	partial VP2	+	3	<3	>236	234 | 77	MK204392.1: 98.7% in aa (blastp)	
	**PBD 05.18 sample**										
12	**Pacific black duck avastrovirus** (PBDAstV)/770nt | PBD05.18 | MT894395	2-26 | Q60	77 | 0.0019%	partial ORF1a	+	1	<1	>768	768 | 255	KJ020899.1: 52.1% in aa (blastp)	pat4 (KKHK) (3) at 125th aa
13	PBDAstV/738nt | PBD05.18 | MT894396	2-18 | Q80	59 | 0.0014%	partial ORF1a	+	2	<2	88	87 | 28		
				partial ORF1b	+	1	76	>738	663 | 220	MN725026.1: 84.6% in aa (blastp)	RdRp at 183-219 aa
14	PBDAstV/1297nt | PBD05.18 | MT894397	2-118 | Q80	294 | 0.0072%	partial ORF2	+	1	13	>1296	1284 | 428	KJ020899.1: 83.1% in aa (MEGA analysis)	Astrovirus capsid protein at 39-378 aa, bipartite (KKKVVKTQSRRGRQRSR) at 9th aa
15	PBDAstV/638nt | PBD05.18 | MT894398	2-20 | Q80	58 | 0.0014%	partial ORF2	+	1	<1	>636	636 | 211	JX624774.1: 42.5% in aa (blastp)	C-terminal tail of astrovirus capsid at 174-207 aa
	**PBD 08.18 sample**										
16	**Pacific black duck orthoreovirus** (PBDORV) segment L1/ 3860nt | PBD08.18 | MT894399	2-336 | Q80	1769 | 0.0150%	partial lambda-A	+	3	6	>3860	3855 | 1284	KC312700.1: 99.0% in aa (blastp)	Inner capsid protein lambda-1 or VP3 at 111-1282 aa, Zinc finger C2H2 type domain at 180-203 aa, pat4 (RRRH) (3) at 8th aa
17	PBDORV segment L2/3661nt | PBD08.18 | MT894400	2-83 | Q80	1079 | 0.0208%	partial lambda-B	+	1	<1	>3660	3660 | 1219	MH520077.1: 98.6% in aa (MEGA analysis)	Reovirus RNA-dependent RNA polymerase lambda 3 at 15-1220 aa, pat4 (RRKH) (3) at 80th aa, pat4 (RRPR) (4) at 521th aa
18	PBDORV segment L3/3624nt | PBD08.18 | MT894401	2-140 | Q80	1917 | 0.0163%	partial lambda-C	+	2	<2	>3622	3621 | 1206	MK955820.1: 97.3% in aa (MEGA analysis)	Reovirus core-spike protein lambda-2 at 2-1206 aa
19	PBDORV segment M1/939nt | PBD08.18 | MT894402	2-64 | Q80	323 | 0.0027%	partial mu-A	+	2	<2	>937	936 | 311	MH520078.1: 97.4% in aa (blastp)	Reovirus minor core protein Mu-2 at 6-205 aa

<table>
<tr><td>20</td><td>PBDORV segment M1/1199nt | PBD08.18 | MT894403</td><td>2-260 | Q80</td><td>704 | 0.0060%</td><td>partial mu-A</td><td>+</td><td>1</td><td><1</td><td>>1197</td><td>1197 | 398</td><td>MH520078.1: 96.7% in aa (blastp)</td><td>Reovirus minor core protein Mu-2 at 1-300 aa, thymidine kinase at 215-362 aa</td></tr>
<tr><td>21</td><td>PBDORV segment M2/497nt | PBD08.18 | MT894404</td><td>2-90 | Q80</td><td>214 | 0.0018%</td><td>partial mu-B</td><td>+</td><td>3</td><td><3</td><td>>497</td><td>495 | 164</td><td>KR476802.1 : 97.5% in aa (blastp)</td><td>Reovirus major virion structural protein Mu-1/Mu-1C (M2) at 2-165 aa</td></tr>
<tr><td>22</td><td>PBDORV segment M2/1066nt | PBD08.18 | MT894405</td><td>2-39 | Q80</td><td>155 | 0.0013%</td><td>partial mu-B</td><td>+</td><td>3</td><td><3</td><td>>1064</td><td>1062 | 353</td><td>KR476802.1 : 99.1% in aa (blastp)</td><td>Reovirus major virion structural protein Mu-1/Mu-1C (M2) at 4-352 aa</td></tr>
<tr><td>23</td><td>PBDORV segment M3/983nt | PBD08.18 | MT894406</td><td>2-35 | Q80</td><td>129 | 0.0011%</td><td>partial mu-NS</td><td>+</td><td>1</td><td>10</td><td>>981</td><td>981 | 326</td><td>MH520080.1: 96.6% in aa (blastp)</td><td></td></tr>
<tr><td>24</td><td>PBDORV segment M3/542nt | PBD08.18 | MT894407</td><td>2-24 | Q80</td><td>76 | 0.0006%</td><td>partial mu-NS</td><td>+</td><td>3</td><td><3</td><td>488</td><td>486 | 161</td><td>MH520080.1: 98.1% in aa (blastp)</td><td></td></tr>
<tr><td>25</td><td>PBDORV segment S1/211nt | PBD08.18 | MT894408</td><td>3-13 | Q80</td><td>376 | 0.0032%</td><td>partial p10</td><td>+</td><td>2</td><td>5</td><td>>211</td><td>207 | 68</td><td>MH520081.1: 67.2% in aa (blastp)</td><td></td></tr>
<tr><td rowspan="2">26</td><td rowspan="2">PBDORV segment S1/1328nt | PBD08.18 | MT894409</td><td rowspan="2">2-67 | Q80</td><td rowspan="2">376 | 0.0032%</td><td>p17</td><td>+</td><td>3</td><td>48</td><td>416</td><td>369 | 122</td><td>JX478256.1: 52.1% in aa (blastp)</td><td rowspan="2">pat7 (PVKRRRL) (5) at 115 on p17. Ribosomal protein L29 at 35-49 aa, 2nd peroxisomal targeting signal (RLHDMDVQL) at 73th aa of sigma C</td></tr>
<tr><td>sigma C</td><td>+</td><td>1</td><td>289</td><td>1314</td><td>1026 | 341</td><td>JX478256.1 and JX145334.1: 54.8% in aa (MEGA analysis)</td></tr>
<tr><td>27</td><td>PBDORV segment S2/1269nt | PBD08.18 | MT894410</td><td>2-30 | Q80</td><td>193 | 0.0016%</td><td>sigma A</td><td>+</td><td>1</td><td>1</td><td>1251</td><td>1251 | 416</td><td>KC508653.1: 97.8% in aa (blastp)</td><td></td></tr>
<tr><td>28</td><td>PBDORV segment S3/1107nt | PBD08.18 | MT894411</td><td>3-173 | Q80</td><td>494 | 0.0042%</td><td>partial sigma B</td><td>+</td><td>3</td><td>30</td><td>>1106</td><td>1077 | 358</td><td>MH520083.1: 96.6% in aa (blastp)</td><td></td></tr>
<tr><td>29</td><td>PBDORV segment S4/884nt | PBD08.18 | MT894412</td><td>2-34 | Q80</td><td>89 | 0.0007%</td><td>partial sigma NS</td><td>+</td><td>3</td><td>9</td><td>>884</td><td>876 | 291</td><td>KJ871025.1: 99.6% in aa (blastp)</td><td></td></tr>
<tr><td>30</td><td>Pacific black duck Rotavirus G (PBDRVG) segment 1/3479nt | PBD08.18 | MT894413</td><td>2-243 | Q80</td><td>1750 | 0.0149%</td><td>partial VP1</td><td>+</td><td>1</td><td><1</td><td>3456</td><td>3456 | 1151</td><td>MK204401: 98.6% in aa (MEGA analysis)</td><td>RdRp of Reoviridae at 537-719 aa</td></tr>
<tr><td>31</td><td>PBDRVG segment 2/2832nt | PBD08.18 | MT894414</td><td>2-309 | Q80</td><td>2439 | 0.0207%</td><td>partial VP2</td><td>+</td><td>2</td><td><2</td><td>2809</td><td>2808 | 935</td><td>MK204402: 98.5% in aa (MEGA analysis)</td><td>AAA-ATPase Vps4-associated protein 1 at 21-81 aa, bipartite (KRVNVKDVDELIAKKEK) at 13th aa, bipartite (KKEDGAAKSDATKKEKD) at 50th aa</td></tr>
<tr><td>32</td><td>PBDRVG segment 4/2277nt | PBD08.18 | MT894415</td><td>2-325 | Q80</td><td>1750 | 0.0149%</td><td>partial VP3</td><td>+</td><td>2</td><td><2</td><td>2245</td><td>2244 | 747</td><td>MK204403: 98.2% in aa (blastp)</td><td></td></tr>
</table>

<table>
<tr><td>33</td><td>PBDRVG segment 3/2322nt | PBD08.18 | MT894416</td><td>2-305 | Q80</td><td>1104 | 0.0213%</td><td>partial VP4</td><td>+</td><td>1</td><td><1</td><td>2304</td><td>2304 | 767</td><td>MF120217.1 and KY689679.1: 48.2% in aa (blastp)</td><td>Rotavirus VP4 membrane interaction domain at 222-335 aa, pat4 (KKKK) (5) at 199th aa, pat7 (PVKKKTS) (5) at 608th aa</td></tr>
<tr><td rowspan="2">34</td><td rowspan="2">PBDRVG segment 5/1198nt | PBD08.18 | MT894417</td><td rowspan="2">2-463 | Q80</td><td rowspan="2">2304 | 0.0136%</td><td>NSP1-1</td><td>+</td><td>2</td><td><2</td><td>325</td><td>324 | 107</td><td></td><td rowspan="2"></td></tr>
<tr><td>NSP1</td><td>+</td><td>3</td><td>183</td><td>1124</td><td>942 | 313</td><td>MK204405: 98.0% in aa (blastp)</td></tr>
<tr><td>35</td><td>PBDRVG segment 6/1272nt | PBD08.18 | MT894418</td><td>2-295 | Q80</td><td>943 | 0.0080%</td><td>VP6</td><td>+</td><td>1</td><td>13</td><td>1194</td><td>1182 | 393</td><td>MK204406: 87.2% in aa (MEGA analysis)</td><td></td></tr>
<tr><td>36</td><td>PBDRVG segment 7/1276nt | PBD08.18 | MT894419</td><td>3-512 | Q80</td><td>1005 | 0.0194%</td><td>NSP3</td><td>+</td><td>2</td><td>50</td><td>1219</td><td>1170 | 389</td><td>MK204407.1 (partial sequence with only 152 aa) : 64.9%; KY689682.1: 44.3% in aa (blastp)</td><td></td></tr>
<tr><td>37</td><td>PBDRVG segment 8/997nt | PBD08.18 | MT894420</td><td>2-139 | Q80</td><td>618 | 0.0052%</td><td>NSP2</td><td>+</td><td>3</td><td>42</td><td>944</td><td>903 | 300</td><td>MK204408: 99.6% in aa (blastp)</td><td>HGXGHXRXV at 229 to 237aa and histidine triad His-X-His-X-His-XX at 229-234aa</td></tr>
<tr><td>38</td><td>PBDRVG segment 9/745nt | PBD08.18 | MT894421</td><td>8-59 | Q80</td><td>114 | 0.0022%</td><td>partial VP7</td><td>+</td><td>3</td><td><3</td><td>695</td><td>693 | 230</td><td>MH453871.1: 51.3% in aa (blastp)</td><td></td></tr>
<tr><td>39</td><td>PBDRVG segment 10/772nt | PBD08.18 | MT894422</td><td>3-126 | Q80</td><td>374 | 0.0031%</td><td>partial NSP4</td><td>+</td><td>2</td><td>131</td><td>>772</td><td>642 | 213</td><td>MH453872.1: 40.9% in aa (blastp)</td><td>pat4 (KKRK) (5) at 67th aa</td></tr>
<tr><td>40</td><td>PBDRVG segment 11/613nt | PBD08.18 | MT894423</td><td>2-311 | Q80</td><td>1750 | 0.0149%</td><td>partial NSP5</td><td>+</td><td>1</td><td><1</td><td>552</td><td>552 | 183</td><td>MK204410: 92.9% in aa (blastp)</td><td></td></tr>
<tr><td rowspan="2"></td><td colspan="11">Chestnut teal</td></tr>
<tr><td colspan="11">CT 08.18 sample</td></tr>
<tr><td>41</td><td>Chestnut teal gammacoronavirus 382nt</td><td>2-9 | Q80</td><td>14 | 0.0001%</td><td>start of ORF1ab</td><td>+</td><td>3</td><td>291</td><td>>380</td><td>90 | 29</td><td>MK204393.1: 100% in nt (blastn)</td><td></td></tr>
<tr><td>42</td><td>Chestnut teal Rotavirus F (CTRVF) segment 7/647nt | CT08.18 | MT894424</td><td>2-11 | Q80</td><td>19 | 0.0001%</td><td>partial NSP3</td><td>+</td><td>3</td><td><3</td><td>>647</td><td>645 | 214</td><td>JQ920001.1: 88.8% in aa (MEGA analysis)</td><td></td></tr>
<tr><td></td><td colspan="11">CT 11.18 sample</td></tr>
<tr><td rowspan="2">43</td><td rowspan="2">Chestnut teal avastrovirus (CTAstV)/1554nt | CT11.18 | MT894425</td><td rowspan="2">3-80 | Q60</td><td rowspan="2">321 | 0.0088%</td><td>partial ORF1a</td><td>+</td><td>1</td><td><1</td><td>1035</td><td>1035 | 344</td><td>MT137996.1: 34.5% in aa (blastp)</td><td rowspan="2">pat4 (PRKR) (4) at 252th aa, pat7 (PLPRKRF) (4) at 250th aa, bipartite (KRGRGRHIHAVKKALRR) at 67th aa</td></tr>
<tr><td>partial ORF1b</td><td>+</td><td>3</td><td>1002</td><td>>1553</td><td>552 | 183</td><td>MH453801.1: 40.0% in aa (blastp)</td></tr>
</table>

44	CTAstV/1564nt | CT11.18 | MT894426	2-195 | Q80	845 | 0.0232%	partial ORF1b	+	3	<3	815	813 | 270	JX985715.1 and JX985714.1: 94.8% in aa (MEGA analysis)	RdRp at 16-147 aa, pat7 (PVRKLKD) (4) at 205th aa of ORF1a. Lysine-rich region at 11.52 aa, Bipartite nuclear localization signal 14-28 aa, pat7 (PRKQKPF) (5) at 17th aa of ORF2.
				partial ORF2	+	2	797	>1564	768 | 256	JX985651.1: 98.0% in aa (MEGA analysis)	
45	CTAstV/344nt | CT11.18 | MT894427	2-25 | Q80	37 | 0.0018%	partial ORF2	+	3	<3	>344	342 | 113	JX985651.1: 99.1% in aa (blastp)	
46	CTAstV/723nt | CT11.18 | MT894428	3-54 | Q80	222 | 0.0112%	partial ORF2	+	2	<2	>721	720 | 239	JX985651.1: 97.08% in aa (blastp)	pat7 (PLQRKDK) (3) at 19th aa
	**Grey teal**										
	**GT 11.18 sample**										
47	**Grey teal gyrovirus** (GTGV) | GT11.18 | MT894388	2-105 | Q80	414 | 0.0147%	VP2	+	3	126	935	810 | 269	MH016741.1: 47.06% in aa (blastp)	pat4 (KKRK) (5) at 140th aa, pat7 (PPPRSKR) (3) at 98th aa, PTPase signature motif CX5R (CSCGNFR) at 46-52 aa the motif WX7HX3CXCX5H at 34-54aa of VP2. Arginine-rich region at 4-68 aa, Bipartite nuclear localization signal at 26-40 aa, the motif WWRWA at 141- 145aa of VP1.
				VP3	+	2	188	589	402 | 133		
				VP1	+	1	668	1534	867 | 288	MH378452.1: 55.2% in aa (MEGA analysis)	

Supplementary material 2

Metagenomic characterisation of additional and novel avian viruses from Australian wild ducks

Running title:

Additional avian DNA and RNA viruses from Australian wild ducks

Supplementary material 2: Genome location of the consensus sequences and phylogenetic analysis of the additional avian viruses in Australian wild ducks

Authors

Jessy Vibin*[1,2], Anthony Chamings[1,2], Marcel Klaassen[3], Soren Alexandersen*[1,2,4]

1 Geelong Centre for Emerging Infectious Diseases, Geelong, VIC 3220, Australia;

2 School of Medicine, Deakin University, Geelong, VIC 3220, Australia;

3 Centre for Integrative Ecology, Deakin University, Waurn Ponds, VIC 3216, Australia;

4 Barwon Health, Geelong, VIC 3220, Australia;

*Corresponding Authors: jessyatall@gmail.com, soren.alexandersen@deakin.edu.au

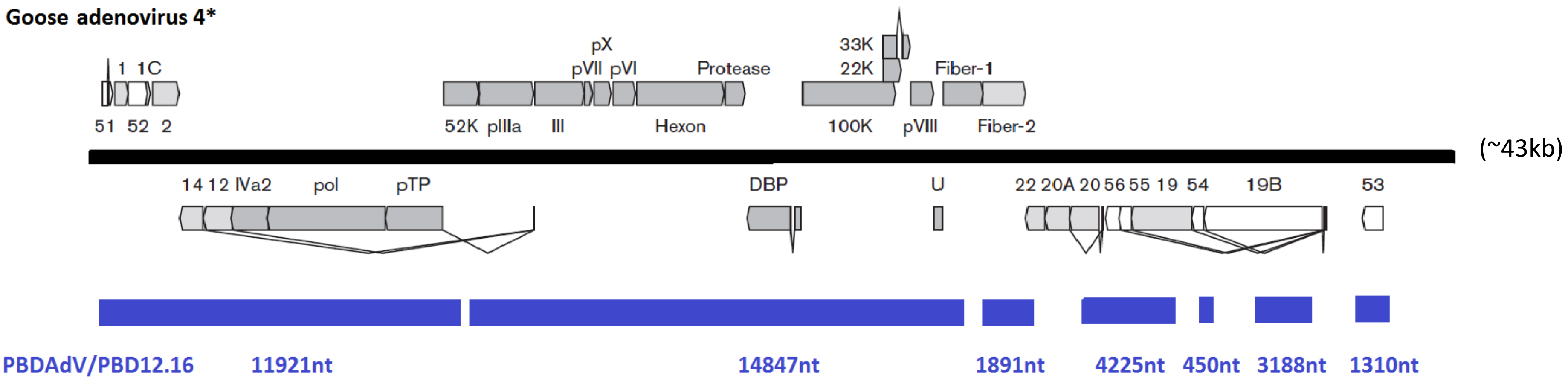

Figure S1: Pacific black duck aviadenovirus (PBDAdV/PBD12.16) consensus sequences aligned to the aviadenovirus reference genome
This figure provides the name of the virus sequence given, the general position of the sequence in the full-length genome of the virus and the proteins encoded by the virus. The reference genome used is JF510462.1 Goose adenovirus 4 (*Adopted and modified from[1]).

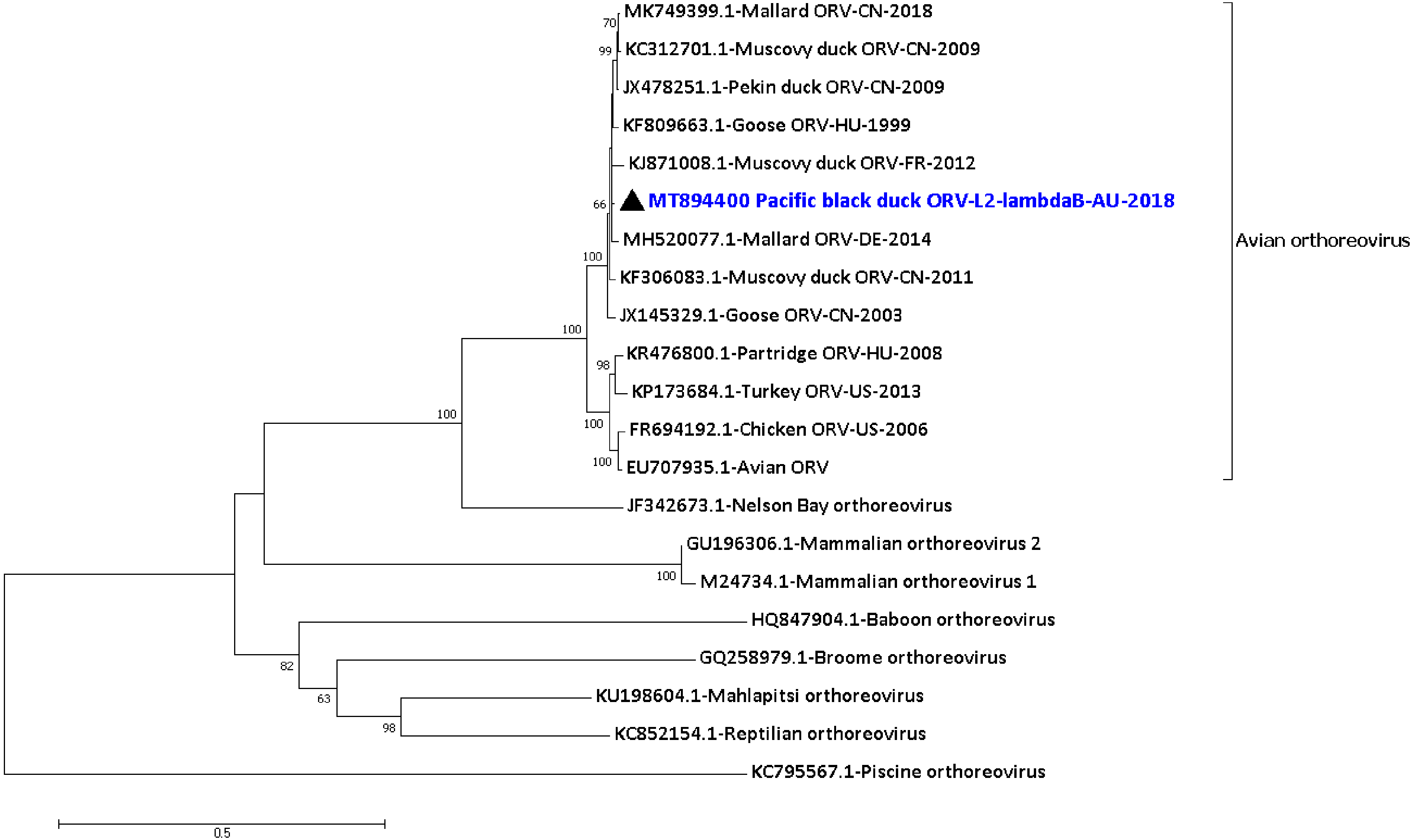

MK749399.1-Mallard ORV-CN-2018
KC312701.1-Muscovy duck ORV-CN-2009
JX478251.1-Pekin duck ORV-CN-2009
KF809663.1-Goose ORV-HU-1999
KJ871008.1-Muscovy duck ORV-FR-2012
MT894400 Pacific black duck ORV-L2-lambdaB-AU-2018
MH520077.1-Mallard ORV-DE-2014
KF306083.1-Muscovy duck ORV-CN-2011
JX145329.1-Goose ORV-CN-2003
KR476800.1-Partridge ORV-HU-2008
KP173684.1-Turkey ORV-US-2013
FR694192.1-Chicken ORV-US-2006
EU707935.1-Avian ORV
JF342673.1-Nelson Bay orthoreovirus
GU196306.1-Mammalian orthoreovirus 2
M24734.1-Mammalian orthoreovirus 1
HQ847904.1-Baboon orthoreovirus
GQ258979.1-Broome orthoreovirus
KU198604.1-Mahlapitsi orthoreovirus
KC852154.1-Reptilian orthoreovirus
KC795567.1-Piscine orthoreovirus
Avian orthoreovirus
70
99
66
100
100
98
100
100
100
100
82
63
98
0.5

Figure S2: Phylogenetic analysis of the amino acid sequences of the lambda B protein of Pacific black duck orthoreovirus segment L2 (PBDORV/L2/PBD08.18)

The amino acid sequences were aligned and analysed by using the maximum likelihood method based on the LG+G+I model[2] in MEGACC[3] with a bootstrapping of 1000 replicates. The analysis involved 21 amino acid sequences and all positions containing gaps and missing data were eliminated. Final dataset contained a total of 1211 amino acid positions. The numbers at the nodes represents bootstrap values and only bootstrap values at or above 60% are shown. Pacific black orthoreovirus is shown in black triangle.

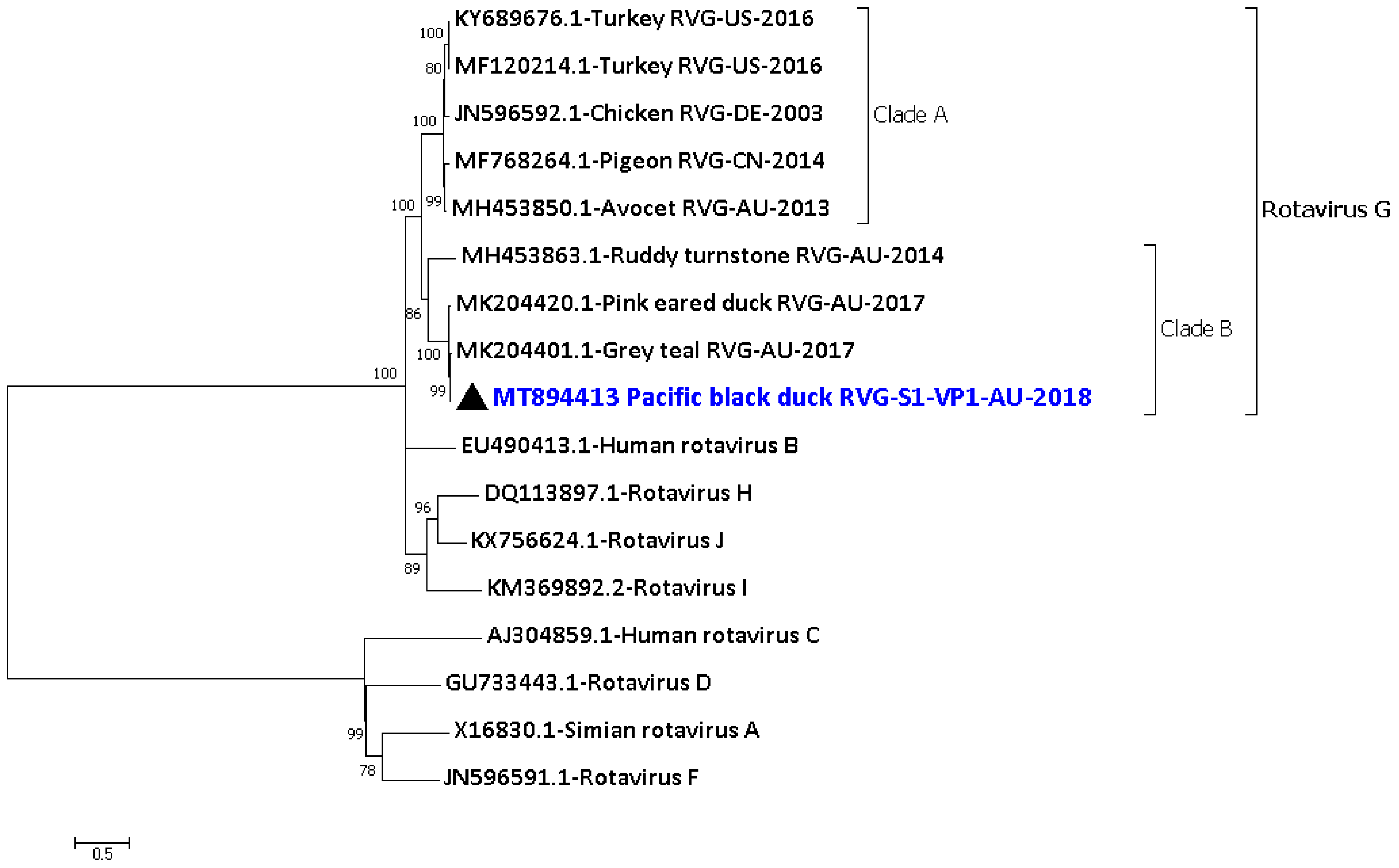
KY689676.1-Turkey RVG-US-2016
MF120214.1-Turkey RVG-US-2016
JN596592.1-Chicken RVG-DE-2003
MF768264.1-Pigeon RVG-CN-2014
MH453850.1-Avocet RVG-AU-2013
MH453863.1-Ruddy turnstone RVG-AU-2014
MK204420.1-Pink eared duck RVG-AU-2017
MK204401.1-Grey teal RVG-AU-2017
MT894413 Pacific black duck RVG-S1-VP1-AU-2018
EU490413.1-Human rotavirus B
DQ113897.1-Rotavirus H
KX756624.1-Rotavirus J
KM369892.2-Rotavirus I
AJ304859.1-Human rotavirus C
GU733443.1-Rotavirus D
X16830.1-Simian rotavirus A
JN596591.1-Rotavirus F
Clade A
Clade B
Rotavirus G
100
80
100
99
100
86
100
99
100
96
89
99
78
0.5

Figure S3: Phylogenetic analysis of the amino acid sequences of the partial VP1 protein of Pacific black duck rotavirus G segment S1 (PBDRVG/S1/PBD08.18)

The amino acid sequences were aligned and analysed by using the maximum likelihood method based on the LG+G+I+F model[2] in MEGACC[3] with a bootstrapping of 1000 replicates. The analysis involved 17 amino acid sequences and all positions containing gaps and missing data were eliminated. Final dataset contained a total of 784 amino acid positions. The numbers at the nodes represents bootstrap values and only bootstrap values at or above 60% are shown. Pacific black rotavirus G is shown in black triangle.

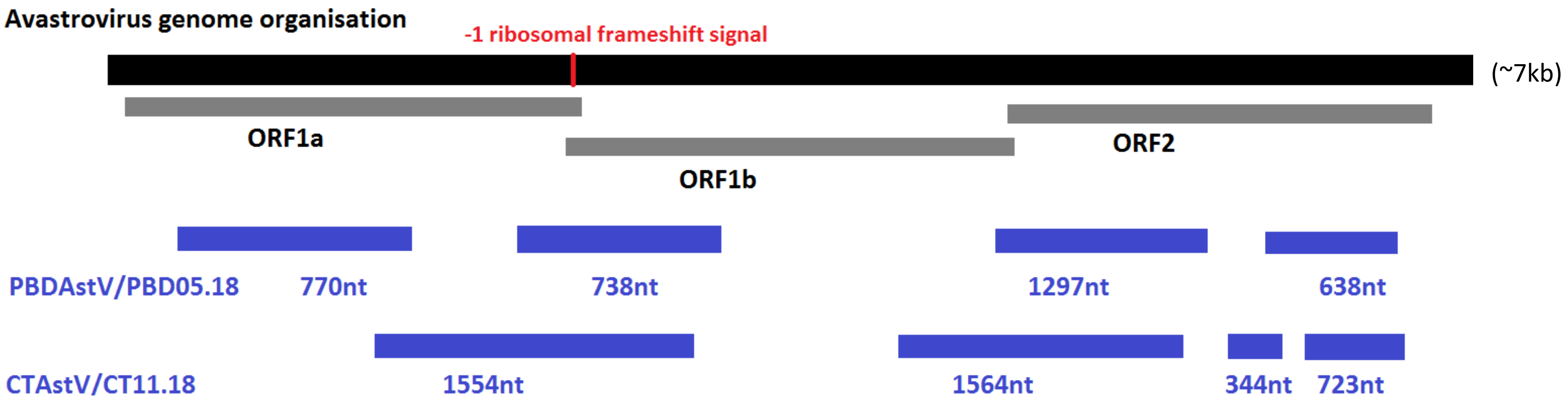

Figure S4: Pacific black duck avastrovirus (PBDAstV/PBD05.18) and Chestnut teal avastrovirus (CTAstV/CT11.18) consensus sequences aligned to the avastrovirus reference genome

This figure provides the name of the virus consensus sequence given, the general position of the consensus sequence in the full-length genome of the virus and the proteins encoded by the virus sequences. Avastrovirus reference genome organisation is given with a note on the -1 ribosomal frameshift signal.

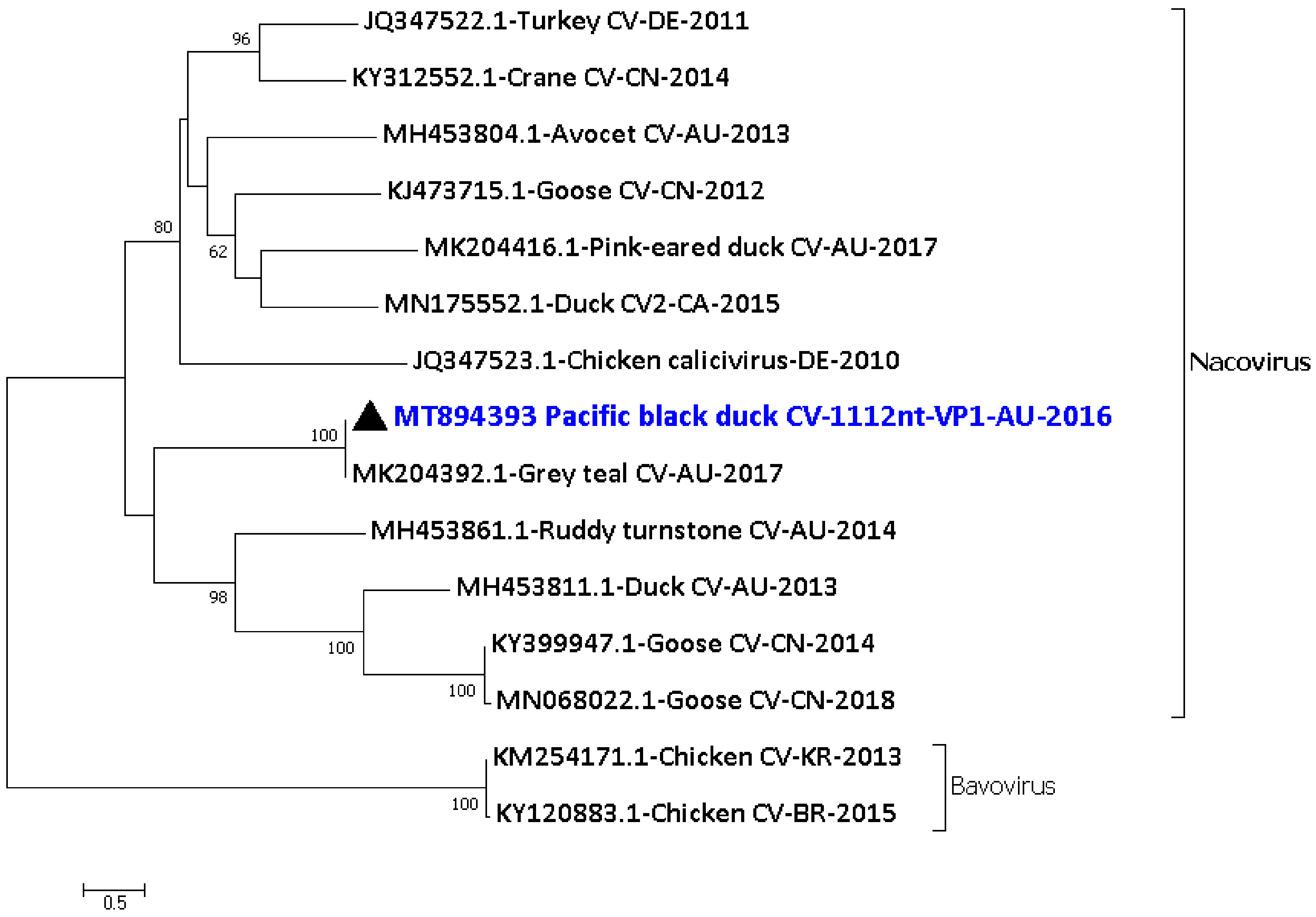
JQ347522.1-Turkey CV-DE-2011
KY312552.1-Crane CV-CN-2014
MH453804.1-Avocet CV-AU-2013
KJ473715.1-Goose CV-CN-2012
MK204416.1-Pink-eared duck CV-AU-2017
MN175552.1-Duck CV2-CA-2015
JQ347523.1-Chicken calicivirus-DE-2010
MT894393 Pacific black duck CV-1112nt-VP1-AU-2016
MK204392.1-Grey teal CV-AU-2017
MH453861.1-Ruddy turnstone CV-AU-2014
MH453811.1-Duck CV-AU-2013
KY399947.1-Goose CV-CN-2014
MN068022.1-Goose CV-CN-2018
KM254171.1-Chicken CV-KR-2013
KY120883.1-Chicken CV-BR-2015
Nacovirus
Bavovirus
96
80
62
100
98
100
100
100
0.5

Figure S5: Phylogenetic analysis of the amino acid sequences of the Pacific black duck calicivirus consensus sequence of 1112 nucleotides long encoding the VP1 protein (PBDCV/1112nt/PBD12.16)

The amino acid sequences were aligned and analysed by using the maximum likelihood method based on the LG+G+F model[2] in MEGA7[4] with a bootstrapping of 1000 replicates. The analysis involved 15 nucleotide sequences and all positions containing gaps and missing data were eliminated. Final dataset contained a total of 329 amino acid positions. The numbers at the nodes represents bootstrap values and only bootstrap values at or above 60% are shown. Pacific black calicivirus is shown in black triangle.

References

1. Kaján, G. L., Davison, A. J., Palya, V., Harrach, B. & Benko, M. Genome sequence of a waterfowl aviadenovirus, goose adenovirus 4. *J. Gen. Virol.* **93**, 2457–2465 (2012).
2. Le, S. Q. & Gascuel, O. An Improved General Amino Acid Replacement Matrix. *Mol. Biol. Evol.* **25**, 1307–1320 (2008).
3. Kumar, S., Stecher, G., Peterson, D. & Tamura, K. MEGA-CC: computing core of molecular evolutionary genetics analysis program for automated and iterative data analysis. *Bioinformatics* **28**, 2685–6 (2012).
4. Kumar, S., Stecher, G. & Tamura, K. MEGA7: Molecular Evolutionary Genetics Analysis Version 7.0 for Bigger Datasets. *Mol. Biol. Evol.* **33**, 1870–4 (2016).
